# Clarifying the structures of imidines: using crystallographic characterization to identify tautomers and localized systems of π-bonding

**DOI:** 10.1107/S2053229623002036

**Published:** 2023-03-15

**Authors:** Michael M. Aristov, Han Geng, James W. Harris, John F. Berry

**Affiliations:** a Department of Chemistry, University of Wisconsin-Madison, 1101 University Ave, Madison, WI 53703, USA; Australian National University, Australia

**Keywords:** organic chemistry, crystal structure, imidine, tautomer, glutarimidine, succinimidine, pyridinone, pyrrolone, historical chemistry

## Abstract

Nitro­gen heterocycles are a class of organic com­pounds with extremely versatile functionality. Imidines, HN[C(NH)*R*]_2_, are a rare class of heterocycles related to imides, HN[C(O)*R*]_2_, in which the O atoms of the carbonyl groups are replaced by N—H groups. The useful synthesis of the imidine com­pounds succinimidine and glutarimidine, as well as their partially hydrolyzed imino–imide congeners, was first described in the mid-1950s, though structural characterization is pre­sented for the first time in this article. In the solid state, these structures are dif­ferent from the proposed imidine form: succinimidine crystallizes as an imino–amine, 2-imino-3,4-di­hydro-2*H*-pyrrol-5-amine, C_4_H_7_N_2_ (**1**), glutarimi­dine as 6-imino-3,4,5,6-tetra­hydro­pyridin-2-amine methanol monosolvate, C_5_H_9_N_3_·CH_3_OH (**2**), and the corresponding hydrolyzed imino–imide com­pounds as amino–amides 5-amino-3,4-di­hydro-2*H*-pyrrol-2-one, C_4_H_6_N_2_O (**3**), and 6-amino-4,5-di­hydro­pyridin-2(3*H*)-one, C_5_H_8_N_2_O (**4**). Imidine **1** was also determined as the hydro­chloride salt solvate 5-amino-3,4-di­hydro-2*H*-pyrrol-2-iminium chloride–2-imino-3,4-di­hydro-2*H*-pyrrol-5-amine–water (1/1/1), C_4_H_8_N_3_^+^·Cl^−^·C_4_H_7_N_3_·H_2_O (**1**·HCl). As such, **1** and **2** show alternating short and long C—N bonds across the mol­ecule, revealing distinct imino (C=NH) and amine (C—NH_2_) groups throughout the C—N backbone. These structures provide definitive evidence for the predominant imino–amine tautomer in the solid state, which serves to enrich the previously proposed imidine-focused structures that have appeared in organic chemistry textbooks since the discovery of this class of com­pounds in 1883.

## Abstract 2

A combination of solid state, solution, and com­putational studies are employed to best describe the various possible tautomers of succinimidine and glutarimidine species and the corresponding hydrolyzed imino–imide com­pounds.

## Introduction

Nitro­gen heterocycles are of considerable inter­est for their ability to act as ligands in coordination chemistry, notably supporting multimetallic com­pounds and, in particular, com­pounds having metal–metal bonds (Chipman & Berry, 2020 ▸; Beach *et al.*, 2021 ▸; Kerru *et al.*, 2020 ▸). Examples of these types of ligands can be seen in 2-naphthyridyl­phenyl­amine (Ding *et al.*, 2015 ▸; Liu, Wang *et al.*, 2009 ▸; Liu, Chen *et al.*, 2009 ▸; Tsai *et al.*, 2013 ▸), 1,8-naphthyridin-2(1*H*)-one (Chang *et al.*, 2017 ▸), 2-anilinopyridinate (Roy *et al.*, 2022 ▸) and 2,2′-di­pyridyl­amine (Hdpa) (Chipman & Berry, 2018*a* ▸,*b* ▸; Lescouëzec *et al.*, 2001 ▸; Berry *et al.*, 2003 ▸; Hsiao *et al.*, 2008 ▸).

We have recently explored the ability of the ligand 2,2′-di­­pyridyl­amine (Scheme 1 shows the structures of Hdpa, succinimide, the proposed ‘succinimidine’ structure, and the observed structure of **1**) to support linear trimetallic metal–metal-bonded com­pounds (Brogden & Berry, 2016 ▸). In the search for other multitopic *N*-donor ligands that might support similar structures, our attention was drawn to the class of com­pounds called ‘imidines’, first described by Pinner in 1883 (Pinner, 1883 ▸) and then later by Elvidge and Linstead in the 1950s. In particular, we focus on the heterocyclic com­pounds ‘succinimidine’ and ‘glutarimidine’ (Elvidge *et al.*, 1959 ▸; Elvidge & Linstead, 1954 ▸). These com­pounds were so named because of their proposed structural analogy to suc­cin­imide (Scheme 1) and the corresponding six-membered-ring analog glutarimide. Since imidines represent a relatively rare functional group, these structures, proposed solely on the basis of elemental analysis results, have been propagated in promi­nent organic chemistry textbooks (March, 1992 ▸). We show here that although solution-based studies agree with the historically predicted imidine tautomers, in the solid state, the com­pounds ‘succinimidine’ and ‘glutarimidine’ adopt a dif­ferent tautomeric form from those originally proposed. In the solid state, the structures are unsymmetric imino–amines and are better named systematically as 2-imino-3,4-di­hydro-2*H*-pyrrol-5-amine (**1**) and 6-imino-3,4,5,6-tetra­hydro­pyridin-2-amine (**2**). 
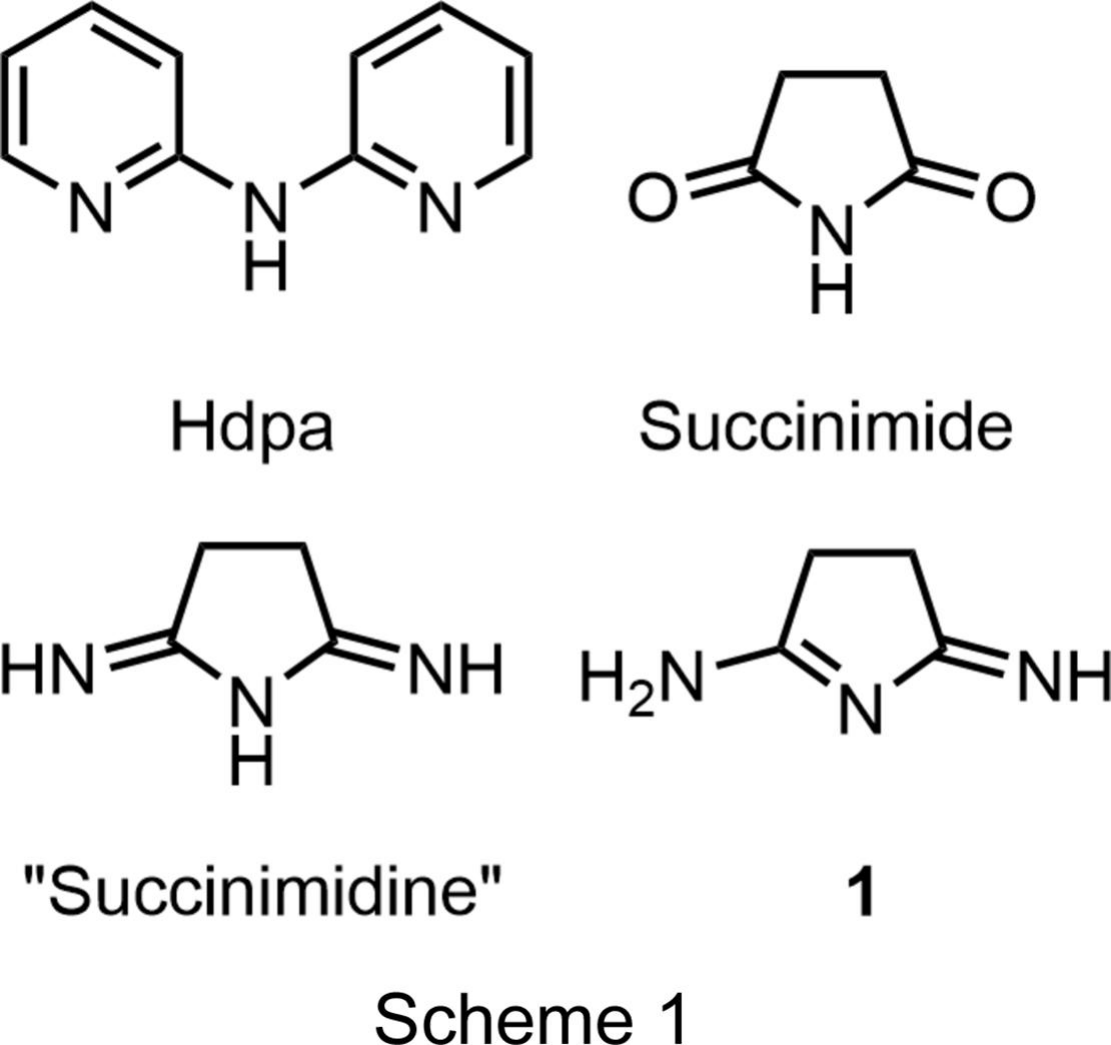


The 1950s syntheses involved the reaction of methanol solutions of terminal di­nitriles (succino­nitrile, glutaro­nitrile, or adipo­nitrile) with liquid ammonia before heating (Elvidge & Linstead, 1954 ▸; Elvidge *et al.*, 1959 ▸). We have found that similar results can be obtained by saturating a methanol solution of succino­nitrile with anhydrous ammonia. This solution, when heated for 18 h in a sealed bomb flask, yielded **1** in >50% yield. The product is easily separated from the mother liquor by precipitation *via* the addition of excess diethyl ether. The synthesis of **2** was performed in an almost identical manner; however, to achieve a useful yield, the reaction mixture was heated for 40 h total. The solvent was then removed by rotary evaporation and yellow crystals separated from the residual oil, which was washed away with ether. The modified Pinner reaction conditions result in protio-neutral ring closing to yield the N-heterocycle with two additional N-atom-based functional groups. Both the original article from Pinner and the later articles from Elvidge and Linstead draw all three N-atom sites as being singly proton­ated in a symmetric ‘imidine’ form (Pinner, 1883 ▸; Elvidge & Linstead, 1954 ▸; Elvidge *et al.*, 1959 ▸). Elvidge and Linstead additionally reported that reaction of the imidines with water sequentially replace one and then both terminal N-atom functional groups with carbonyl groups, such that ‘succinimidine’ could be fully hydrolyzed to form succinimide (Elvidge & Linstead, 1954 ▸; Elvidge *et al.*, 1959 ▸). While the symmetric structure of succinimide in the solid state is well established (Yu *et al.*, 2012 ▸; Mason, 1961 ▸), the mono­hydrolyzed forms of **1** and **2** have not been investigated before, and they are structurally characterized here (Scheme 2 shows the structures of the most stable solid-phase tautomers of the species described . 
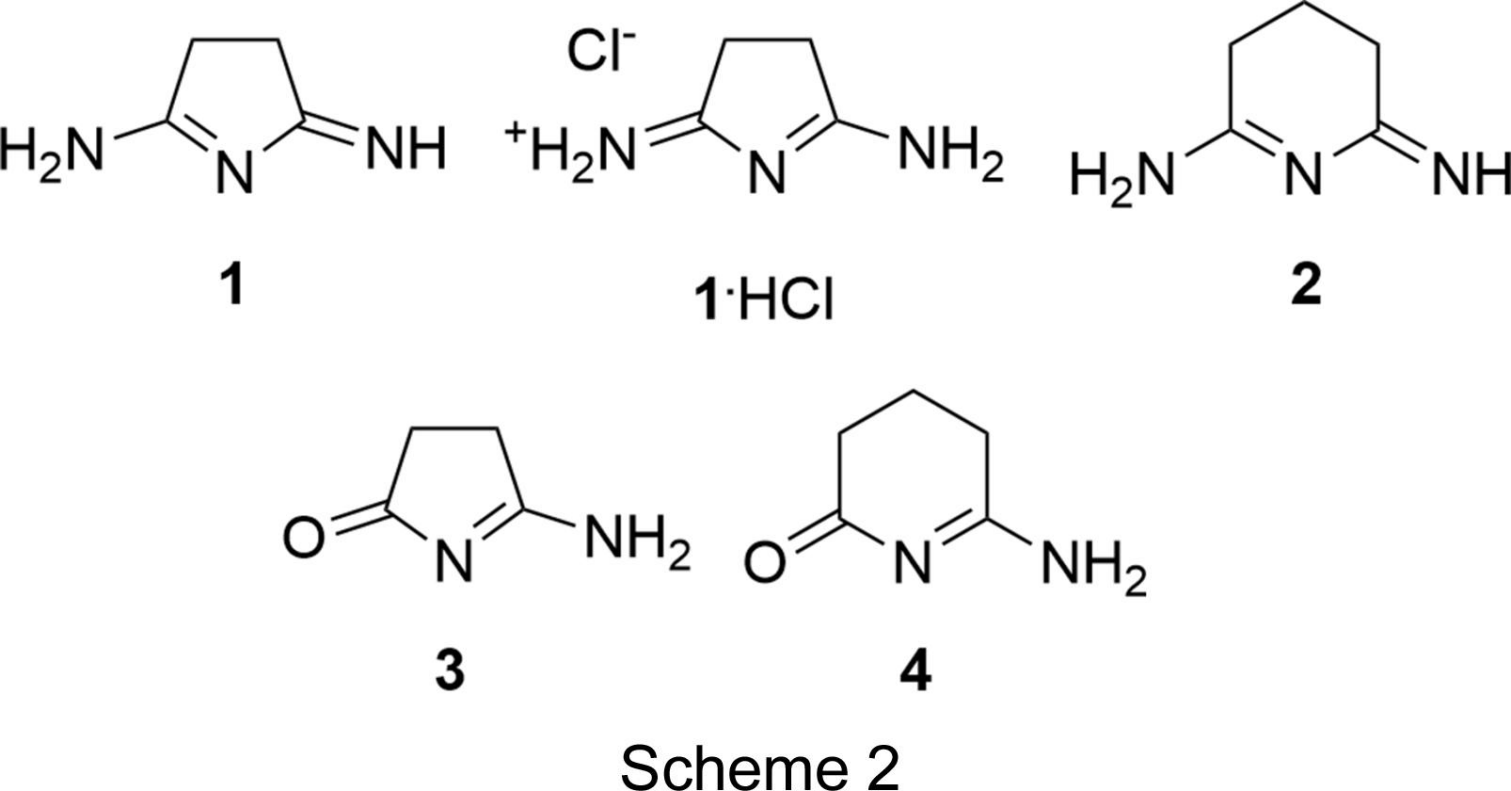
 in this article, with only one resonance structure being shown for the protonated species found in **1**·HCl). A combination of solid state, solution, and com­putational studies are employed to best describe the various possible tautomers of these species.

## Experimental

### General methods

Methanol (Sigma–Aldrich) was distilled from CaH_2_ under N_2_ and used immediately. Succino­nitrile and glutaro­nitrile were purchased from Sigma–Aldrich and used as received. Inhibitor-free anhydrous diethyl ether was purchased from Sigma–Aldrich and used as received. All deuterated solvents were purchased from Sigma–Aldrich, used as received, and stored long term in air. Unless otherwise noted, all mani­pulations were performed in air. Electrospray ionization mass spectrometry was performed with a Thermo Q Exactive Plus mass spectrometer. IR spectra were recorded with a Bruker Tenser 27 spectrometer using an ATR adapter. ^1^H NMR spectra were recorded on a 400 MHz Bruker Avance III spectrometer. **Caution!** The synthetic procedures for the preparation of **1** and **2** involve heating a sealed reaction vessel and should only be performed at or below the scale described here using rated thick-walled glassware, with a protective blast shield.

### Synthesis and crystallization

#### Synthesis of 1

Imidine **1** was synthesized through a modification of the literature procedure of Elvidge & Linstead (1954 ▸). Anhydrous methanol (70 ml), succino­nitrile (4.02 g, 50.1 mmol), and a Teflon stirrer bar were combined in a 250 ml heavy-walled threaded glass vessel. The solid was fully dis­solved and the resulting solution was sparged with anhydrous ammonia gas until saturated. The flask was then tightly sealed and partially submerged in an oil bath. The oil bath was programmed to heat to 70 °C for 18 h before automatically cooling to room temperature. A blast shield was placed in front of the flask and the heating cycle was started. Upon cooling to room temperature, the pressure flask containing a black solution was removed from the oil bath. Activated carbon (∼3 g) was added to the solution, which was sparged with nitro­gen for 10 min. The solution was then filtered through Celite to yield a pale-yellow filtrate. This filtrate was added to diethyl ether (300 ml), resulting in precipitation of the product. The suspension was filtered through a glass frit and the off-white solid was washed several times with ether. The solid was dried under high-vacuum overnight and stored in a nitro­gen glove-box without further purification. X-ray-quality crystals were obtained by slow diffusion of diethyl ether into a saturated solution of **1** in MeOH under an inert atmosphere. ESI (*m*/*z*): ([*M* + H]^+^) 98.0712. IR (ATR, cm^−1^): 3289, 3157, 3077, 2935, 2847, 1829, 1772, 1749, 1686, 1662, 1654, 1636, 1532, 1473, 1453, 1418, 1328, 1296, 1265, 1241, 1223, 1190, 1143, 1129, 1115, 996, 936, 919, 851, 822, 783, 665, 651, 641. ^1^H NMR (400 MHz, DMSO): δ 7.37 (*s*, 3H), 2.46 (*s*, 4H). Crystals of **1**·HCl were fortuitously obtained by slow diffusion of diethyl ether into a deuterated chloro­form solution containing **1** (yield: 2.46 g, 25.3 mmol, 50.6%).

#### Synthesis of 2

Imidine **2** was synthesized by a modified literature method (Elvidge & Linstead, 1954 ▸). Anhydrous methanol (70 ml), glutaro­nitrile (2.0299 g, 21.568 mmol), and an oven-dried stirrer bar were added to an oven-dried pressure flask under a constant stream of nitro­gen gas. The resulting solution was sparged with nitro­gen gas for 5 min and then saturated with ammonia gas. The flask was then sealed and heated at 70 °C for 40 h while stirring. Once the flask had cooled, the clear solution was sparged with nitro­gen for ∼20 min. The solvent was removed *via* rotary evaporation. The resulting yellow powder was washed with diethyl ether and filtered to remove residual glutaro­nitrile. X-ray-quality crystals were obtained by evaporation of a saturated MeOH solution (yield: 0.760 g, 31.7%). ESI (*m*/*z*): ([*M* + H]^+^) 112.0868. IR (ATR, cm^−1^): 3254, 3004, 2954, 1666, 1605, 1543, 1457, 1418, 1373, 1334, 1316, 1316, 1187, 1145, 1103, 1061, 967, 909, 886, 791, 758, 676. ^1^H NMR (400 MHz, DMSO): δ 7.05 (*s*, 3H), 2.20 (*t*, *J* = 6.5 Hz, 4H), 1.80–1.57 (*q*, 2H).

#### Synthesis of 3

A scintillation vial was charged with **1** (1.0 g, 0.010 mol). Milli-Q water (3.4 ml, 0 °C) was then added to the vial, immediately turning the solution faint brown. The vial was stored in a 0 °C refrigerator overnight. The next day, white crystals (yield: 0.68 g, 0.0069 mol, 69%) suitable for X-ray diffraction analysis were collected from the solution. ESI (*m*/*z*): ([*M* + H]^+^) 99.0552. IR (ATR, cm^−1^): 3220, 3135, 3019, 2938, 2918, 2851, 2360, 2341, 1686, 1627, 1526, 1456, 1437, 1418, 1397, 1338, 1294, 1251, 1221, 1161, 1009, 929, 866, 852, 827, 765, 677. ^1^H NMR (400 MHz, DMSO): δ 8.30 (*s*, 1H), 8.07 (*s*, 1H), 2.67–2.56 (*m*, 2H), 2.34–2.25 (*m*, 2H).

#### Synthesis of 4

A scintillation vial was filled with **2** (0.10 g, 0.90 mmol) and the solid was subsequently dissolved in a minimal amount of Milli-Q water. The resulting solution was cooled overnight before allowing ether vapor to diffuse into the solution. The product precipitated out as white crystals (yield 0.048 g, 47%) suitable for X-ray diffraction, with a minor impurity of 6-hy­droxy-4,5-di­hydro­pyridin-2(3*H*)-one. ESI (*m*/*z*): ([*M* + NH_4_]^+^): 130.0975. IR (ATR, cm^−1^): 3381, 3185, 2967, 2947, 2920, 2886, 2823, 2774, 1644, 1534, 1506, 1458, 1426, 1418, 1349, 1299, 1274, 1222, 1153, 1120, 1071, 1056, 948, 917, 864, 807, 756, 671, 638. ^1^H NMR (400 MHz, DMSO-*d*_6_): δ, 7.35 (*s*, 1H), 6.80 (*s*, 1H), 2.24 (*t*, *J* = 7.7 Hz, 2H), 1.88 (*t*, *J* = 7.3 Hz, 2H), 1.78 (quint, *J* = 7.4 Hz, 2H).

### Refinement

Crystal data, data collection and structure refinement details are summarized in Table 1 ▸. For the structures of **1** and **4**, the diffraction data were consistent with a triclinic unit cell. The *E*-statistics for **1** and **4** strongly suggested the centrosymmetric space group *P* 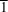 , which yielded chemically reasonable and com­putationally stable refinements. For the structures of **2**, **3**, and **1**·HCl, a combination of systematic absences in the diffraction data and the *E*-statistics were used to assign the centrosymmetric space groups *P*2_1_/*c*, *P*2_1_/*n*, and *C*2/*c*, respectively.

The structures were solved *via* intrinsic phasing and refined by least-squares refinement on *F* ^2^, followed by difference Fourier synthesis. All non-H atoms above 70% occupancy were refined with anisotropic displacement parameters. Unless otherwise stated, all H atoms were included in the final structure-factor calculations at idealized positions and were allowed to ride on their neighboring atoms with relative isotropic displacement coefficients. In the structure **1**·HCl, all amine H atoms were fixed at idealized locations, where as the imidine and water H atoms were allowed to freely refine.

The coordinates of the H atoms bound to N atoms in **1**, **3**, and **4** were allowed to refine freely. In **2**, residual electron density provided strong evidence for the coordinates of the N-atom-bound H atoms; however, there was not sufficient electron density to allow the H atoms to refine freely. As such, the coordinates of the H atoms bound to N atoms in **2** were fixed at idealized positions.

In the structure of **2**, the three methyl­ene C atoms of the ring are disordered over two positions, with a major occupancy of 85.4 (6)%. The lesser fraction of the disordered part of the ring was restrained to the geometry of the major fraction of the same ring. One of the methanol solvent mol­ecules exhibited disorder of the CH_3_ protons.

## Results and discussion

### Structural commentary

**Figure 1:**
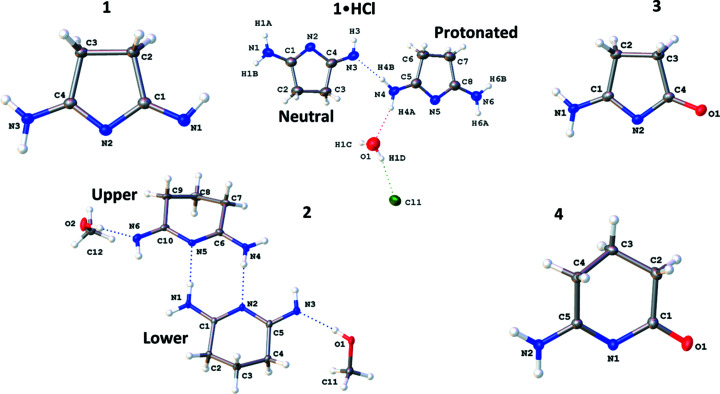
The asymmetric units of **1** (top left), **1**·HCl (top middle), **3** (top right), **2** (bottom left) and **4** (bottom right), shown with 50% probability displacement ellipsoids. Dotted lines are used to indicate hydro­gen-bonding inter­actions. Only the major disorder com­ponent of the ring in **2** is shown. Additional labels for **1**·HCl and **2** are included for clarification in later discussion.

Three of the title N-heterocycles, namely, **1**, **3**, and **4**, crystalize with only one mol­ecule in the asymmetric unit, with no disorder or solvent mol­ecules. The crystal structure for **2** includes two N-heterocycles and two methanol solvent mol­ecules in the asymmetric unit. The two independent mol­ecules of **2** (denoted ‘upper’ and ‘lower’) inter­act *via* a set of two N—H⋯N hydro­gen bonds to form a dimeric structure. A similar structural motif is seen in the structure of succinimide (Yu *et al.*, 2012 ▸; Mason, 1961 ▸) and for some of the other com­pounds described here, when looking at the structures beyond just the asymmetric unit (*vide infra*). Additionally, one of the mol­ecules of **2** displays disorder across the three –CH_2_– units in the backbone, and one methanol mol­ecule shows disorder of the H atoms on the –CH_3_ group. The structure of **1**·HCl contains one neutral five-membered heterocycle, its protonated species, a Cl^−^ counter-ion, and one solvent water mol­ecule. The asymmetric unit of each structure is shown in Fig. 1 ▸.

In **1**, the NH protons are distributed such that one terminal N atom is doubly protonated as an amine, the N atom in the ring is not protonated, and the other terminal N atom is singly protonated, as an imine, with the proton pointing towards the hydro­phobic backbone. In **2**, the H atoms are distributed in a nearly identical manner. However, due to inter­molecular O—H⋯N hydro­gen-bonding inter­actions with the solvent methanol mol­ecules, the imine N atom of each of the two independent mol­ecules of **2** has its single H atom pointed away from the hydro­phobic backbone. In both **3** and **4**, the O atom binds as a carbonyl group, as indicated by the short C=O distances of 1.231 (1) and 1.238 (1) Å. As in the NNN structures, the N atom in the ring is not protonated, and the terminal N atom is doubly protonated as an amine. Crystals of **1**·HCl were obtained fortuitously from slow diffusion of diethyl ether into a solution of deuterated chloro­form containing **1**. In the structure of **1**·HCl, there exists both a neutral species, com­parable to the heterocycle found in **1**, and a protonated cationic species where both terminal N atoms are doubly protonated, with the N atom in the ring being left unprotonated. The protonated species in **1**·HCl is balanced by a Cl^−^ anion. The protonation states of all the com­plexes can be seen in Fig. 1 ▸. Notably, the protonation states of all the com­pounds differ from the structure of succinimide, which remains symmetric despite forming similarly asymmetric hydro­gen-bonded dimers (Yu *et al.*, 2012 ▸; Mason, 1961 ▸). The structures of **1** and **2** are also notably inconsistent with their earlier structural proposals as ‘succinimidine’ and ‘glutarimidine’, and it is particularly notable that protonation of **1** to form the HCl salt occurs at a terminal imine rather than the inter­nal ring position. These observations are consistent with p*K_a_* data for terminal *versus* inter­nal imines: (Ph)_2_C=NH (p*K_a_* = 31.0) (Bordwell & Ji, 1991 ▸) and PhCH_2_N=C(Ph)_2_ (p*K_a_* = 24.3) (Bordwell, 1988 ▸).

**Figure 2:**
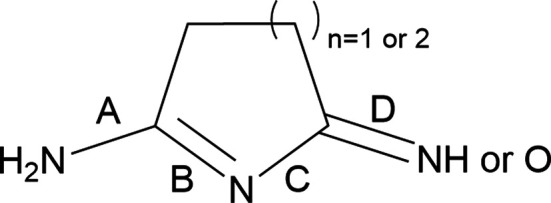
A generic structure used to define the bonds of inter­est.

The proposed protonation states of **1**–**4** are further supported by the bond lengths across the heteroatoms, as seen in Fig. 2 ▸. These bond distances, as well as relevant com­parisons, are given in Table 2 ▸. We note the neutral com­pounds show statistically meaningful differences between the A/D and B/C bond pairs defined in Fig. 2 ▸. Specifically, these differences appear to indicate a localized π-system with alternating single and double bonds, where the shorter bonds are localized to B and D. In contrast, these differences in the structure of the protonated species of **1**·HCl are statistically insignificant. Thus, the structure of the protonated species in **1**·HCl is best described by a delocalized electronic structure which could be represented by the two limiting resonance forms shown in Scheme 3. Notably, the neutral species in **1**·HCl shows nearly identical differences in the bond lengths to those in **1**. Notably, the neutral molecule in **1**·HCl and in **2** show an alternate binding motif for the imine-bound proton observed in **1**. This alternative binding motif likely arises from the hydrogen-bonding interaction blocking the other side of the imine. 
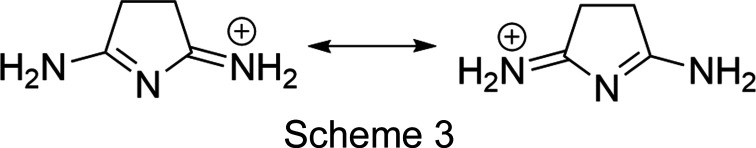


To gain further insights into the protonation states of **1**, com­putational studies were performed. All calculations were carried out using *GAUSSIAN16* (Frisch *et al.*, 2016 ▸), Hartree–Fock theory, and the 6-31g(d) basis set. Input geometries were constructed from modified crystallographic coordinates. The geometry-optimized *xyz* coordinates for all structures are provided in the supporting information (Tables S1 and S2). The calculations indicate that, in the gas phase, the Gibbs free energy of the symmetric ‘succinimidine’ tautomer is ∼1.9 kcal mol^−1^ more stable than the asymmetric form observed crystallographically. The energy difference is small enough to allow for the network of hydro­gen bonds in the crystal structure to dictate which tautomer of the com­pound is observed in the solid state. This packing-influenced tautomerization also aligns with previous tautomer-based studies that utilized variable-temperature crystallography and ther­mal evolution to better understand the tautomer ratios in keto–amine/imino­enol systems (Godsi *et al.*, 2004 ▸). To examine which tautomer is preferred in solution, we examined a solution of **1** in DMSO-*d*_6_ by ^1^H NMR spectroscopy. The main signal observed is a singlet at 2.46 ppm assignable to the CH_2_ protons, consistent with the symmetric ‘succinimidine’ tautomer. This provides evidence that in solution, the imidine structure, as historically drawn in textbooks (March, 1992 ▸), dominates, yet in the solid state, the asymmetric tautomer is prevalent. Additionally, the singlet at 7.37 ppm likely indicates rapid exchange between all three of the NH protons. For reference, the ^1^H NMR spectrum of succinimide in CDCl_3_-*d*_1_ consists of a singlet at 2.769 ppm (https://www.chemicalbook.com/SpectrumEN_123-56-8_1HNMR.htm).

### Crystal packing

**Figure 3:**
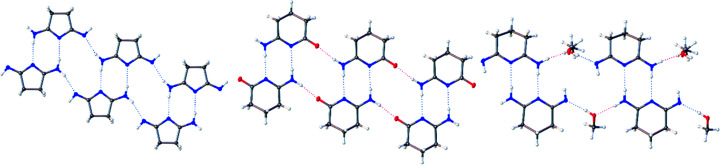
A com­parison of the planar dimers formed by **1** (left), **4** (middle) and **2** (right), shown with 50% probability displacement ellipsoids. Dotted lines are used to indicate hydro­gen-bonding inter­actions. Only the major disorder com­ponent of the ring in **2** is shown.

**Figure 4:**
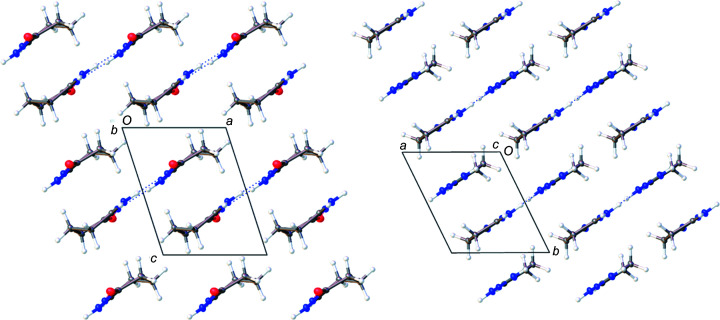
(Left) A mol­ecular drawing of **4**, viewed along the crystallographic *b* axis. (Right) A mol­ecular drawing of **1**, viewed along the crystallographic *c* axis. Both structures are drawn with 50% probability displacement ellipsoids. Dotted lines are used to indicate hydro­gen-bonding inter­actions.

**Figure 5:**
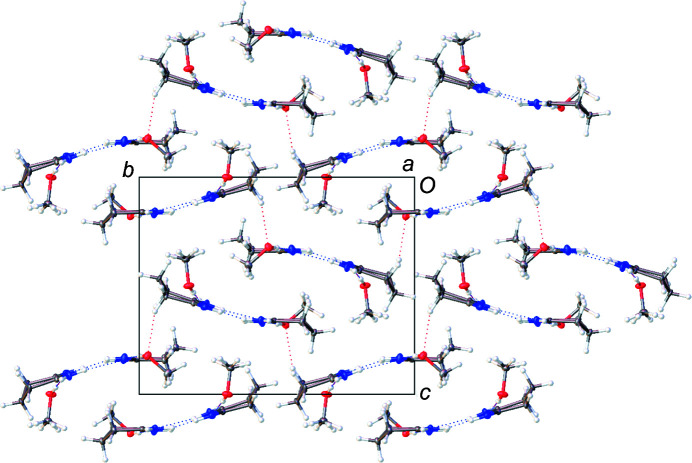
A mol­ecular drawing of **2**, viewed along the crystallographic *a* axis, drawn with 50% probability displacement ellipsoids. Dotted lines are used to indicate hydro­gen-bonding inter­actions.

Unsurprisingly, the large number of hydro­gen-bond donors and acceptors in the mol­ecules examined here result in significant inter­molecular hydro­gen-bonding inter­actions throughout the crystal structures (Tables 3 ▸–7 ▸ ▸ ▸ ▸). In **1**, **2**, and **4**, the hydro­gen-bonding inter­actions result in oligomerization of the planar dimer units formed by the hydro­philic section of the mol­ecules being paired together (Fig. 3 ▸). Each pair involves a double-hydro­gen-bonded eight-membered ring reminiscent of the structural motifs seen for carb­oxy­lic acid dimers in the solid (Jasinski *et al.*, 2009 ▸), solution (Kolbe *et al.*, 1997 ▸), or gas phase (Emmeluth *et al.*, 2003 ▸). The linking of these hydro­gen-bonded dimers through further lateral hy­dro­gen bonds creates long two-dimensional ribbons through­out the crystal lattice. These ribbons stack together to form the three-dimensional crystal structures. For both **1** and **4**, there are no hydro­gen-bonding inter­actions between ribbons either in the same plane or in between planes, as seen in Fig. 4 ▸. This pattern is broken with **2**, where the methanol solvent mol­ecule hydro­gen bonds in between sheets. This additional hydro­gen-bonding inter­action perpetuates through­out the packed crystal structure, making a series of inter­laced sheets, as seen in Fig. 5 ▸. Com­pounds **1** and **2** contain a mismatch in the number of hydro­gen-bond-donating and -accepting groups, leading to structures in which one of the potential hydro­gen-bond donors remains unsatisfied.

**Figure 6:**
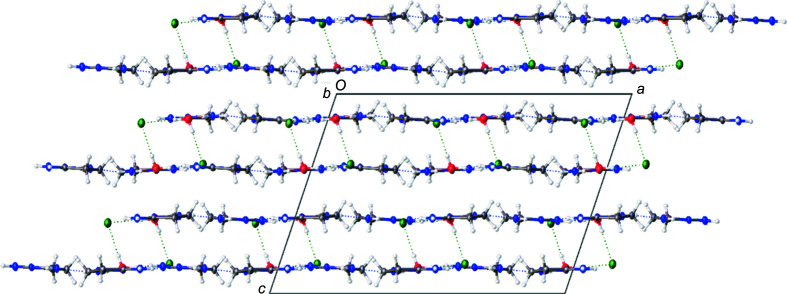
A molecular drawing of **1**·HCl, viewed along the crystallographic *b* axis, shown with 50% probability displacement ellipsoids. Dotted lines are used to indicate hydrogen-bonding interactions. The figure depicts hydrogen-bonding interactions between sheets, bridging pairs of these sheets.

**Figure 7:**
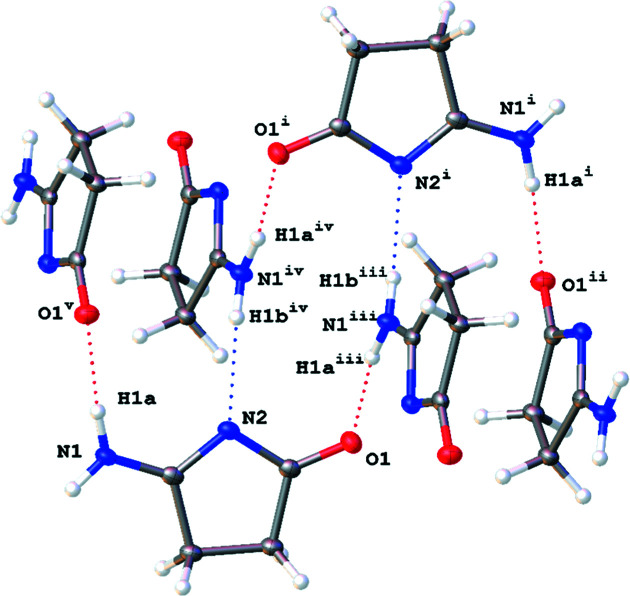
The stacked hydro­gen-bonding network observed in **3**. All atoms are drawn with 50% probability displacement ellipsoids and dotted lines are used to indicate hydro­gen-bonding inter­actions. [Symmetry codes: (i) *x* + 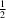 , −*y* + 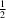 , *z* + 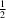 ; (ii) −*x* + 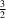 , *y* + 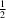 , −*z* + 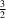 ; (iii) −*x* + 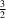 , *y* − 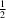 , −*z* + 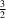 ; (iv) −*x* + 2, −*y* + 1, −*z* + 2; (v) *x* + 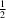 , −*y* + 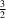 , *z* + 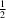 .]

In the structure of **1**·HCl, hydro­phobic backbone and hydro­philic heteroatoms alternate in the plane, as seen in Fig. 1 ▸. Additionally, the solvent water mol­ecule in **1**·HCl hydro­gen bonds between sheets, bridging pairs of these sheets, as seen in Fig. 6 ▸. The major exception to the planar mol­ecular sheets stabilized by a hydro­gen-bond network is found in the crystal packing of **3**. Compound **3** does not form discrete carb­oxy­lic acid-style dimers. Instead, each mol­ecule of **3** has hydro­gen-bonding inter­actions with four other mol­ecules of **3** that form an inter­connected three-dimensional lattice as the mol­ecules stack perpendicular to each other, as seen in Fig. 7 ▸. The introduction of the three-dimensional hydro­gen-bonding lattice is likely what aids in the crystallization of **3** from aqueous conditions.

## Summary

Through careful analysis of solid-state and solution phase measurements of the historical imidines, the apparent experimental disagreement between whether their structures are asymmetric or symmetric tautomers has been resolved. The crystallographic data provide evidence for the solid-state asymmetric tautomer for both the five- and six-membered ring compounds, whereas solution phase NMR spectroscopy data strongly indicate a more symmetric form. The energetic differences between the symmetric and asymmetric forms were calculated to be sufficiently small to allow for tautomerization to reasonably occur in solution at room temperature. The synthetic methods and characterization of these compounds have been modernized and safety issues associated with the synthesis have been clarified.

## Supplementary Material

Crystal structure: contains datablock(s) 1HCl, 1, 3, 4, 2, global. DOI: 10.1107/S2053229623002036/ep3031sup1.cif

Structure factors: contains datablock(s) 1HCl. DOI: 10.1107/S2053229623002036/ep30311HClsup2.hkl

Structure factors: contains datablock(s) 1. DOI: 10.1107/S2053229623002036/ep30311sup3.hkl

Structure factors: contains datablock(s) 3. DOI: 10.1107/S2053229623002036/ep30313sup5.hkl

Structure factors: contains datablock(s) 4. DOI: 10.1107/S2053229623002036/ep30314sup6.hkl

Structure factors: contains datablock(s) 2. DOI: 10.1107/S2053229623002036/ep30312sup4.hkl

Supporting information file. DOI: 10.1107/S2053229623002036/ep30311HClsup7.cml

Supporting information file. DOI: 10.1107/S2053229623002036/ep30311sup8.cml

Supporting information file. DOI: 10.1107/S2053229623002036/ep30313sup9.cml

Supporting information file. DOI: 10.1107/S2053229623002036/ep30314sup10.cml

Supporting information file. DOI: 10.1107/S2053229623002036/ep30312sup11.cml

Additional spectra, figures and tables. DOI: 10.1107/S2053229623002036/ep3031sup12.pdf

CCDC references: 2202458, 2202457, 2202456, 2202455, 2202454

## Figures and Tables

**Table:** Experiments were carried out at 100 K using a Bruker SMART APEXII (Quasar) diffractometer. H atoms were treated by a mixture of independent and constrained refinement. Absorption was corrected for by multi-scan methods (*SADABS*; Bruker, 2016 ▸; Krause *et al.*, 2015 ▸).

	**1**	**1**·HCl	**2**
Crystal data			
Chemical formula	C_4_H_7_N_3_	C_4_H_8_N_3_^+^·Cl^−^·C_4_H_7_N_3_·H_2_O	C_5_H_9_N_3_·CH_4_O
*M*_r_	97.13	248.72	143.19
Crystal system, space group	Triclinic, *P* 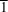	Monoclinic, *C*2/*c*	Monoclinic, *P*2_1_/*c*
*a*, *b*, *c* (Å)	5.9577 (4), 6.7494 (5), 6.8249 (5)	19.294 (3), 9.4173 (8), 13.7430 (12)	9.4887 (9), 14.5341 (11), 12.2828 (10)
α, β, γ (°)	101.641 (4), 104.225 (6), 111.425 (4)	90, 108.570 (5), 90	90, 111.320 (8), 90
*V* (Å^3^)	234.36 (3)	2367.0 (5)	1578.0 (2)
*Z*	2	8	8
Radiation type	Cu *K*α	Cu *K*α	Cu *K*α
μ (mm^−1^)	0.75	2.81	0.70
Crystal size (mm)	0.11 × 0.11 × 0.10	0.09 × 0.04 × 0.04	0.03 × 0.02 × 0.01

Data collection			
*T*_min_, *T*_max_	0.844, 0.901	0.852, 0.947	0.690, 0.754
No. of measured, independent and observed [*I* > 2σ(*I*)] reflections	3673, 892, 833	19987, 2322, 1934	26582, 3219, 2938
*R*_int_	0.019	0.051	0.037
(sin θ/λ)_max_ (Å^−1^)	0.618	0.621	0.625

Refinement			
*R*[*F* ^2^ > 2σ(*F* ^2^)], *wR*(*F* ^2^), *S*	0.034, 0.092, 1.09	0.044, 0.116, 1.03	0.040, 0.108, 1.07
No. of reflections	892	2322	3219
No. of parameters	76	151	204
No. of restraints	0	0	5
Δρ_max_, Δρ_min_ (e Å^−3^)	0.27, −0.27	0.44, −0.24	0.35, −0.28

**Table:** 

	**3**	**4**
Crystal data		
Chemical formula	C_4_H_6_N_2_O	C_5_H_8_N_2_O
*M*_r_	98.11	112.13
Crystal system, space group	Monoclinic, *P*2_1_/*n*	Triclinic, *P* 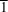
*a*, *b*, *c* (Å)	7.3685 (5), 8.0074 (7), 8.4211 (9)	6.3296 (19), 7.0222 (19), 7.351 (2)
α, β, γ (°)	90, 115.741 (5), 90	84.975 (13), 71.693 (13), 63.889 (12)
*V* (Å^3^)	447.56 (7)	278.06 (14)
*Z*	4	2
Radiation type	Cu *K*α	Mo *K*α
μ (mm^−1^)	0.91	0.10
Crystal size (mm)	0.1 × 0.09 × 0.04	0.16 × 0.05 × 0.01

Data collection		
*T*_min_, *T*_max_	0.853, 0.915	0.929, 0.991
No. of measured, independent and observed [*I* > 2σ(*I*)] reflections	7398, 886, 775	9023, 2048, 1680
*R*_int_	0.042	0.034
(sin θ/λ)_max_ (Å^−1^)	0.617	0.770

Refinement		
*R*[*F* ^2^ > 2σ(*F* ^2^)], *wR*(*F* ^2^), *S*	0.033, 0.089, 1.03	0.043, 0.120, 1.06
No. of reflections	886	2048
No. of parameters	72	79
No. of restraints	0	0
Δρ_max_, Δρ_min_ (e Å^−3^)	0.23, −0.18	0.44, −0.27

Computer programs: *APEX3* (Bruker, 2016 ▸, 2017 ▸), *SAINT-Plus* (Bruker, 2016 ▸), *SAINT* (Bruker, 2017 ▸), *olex2.solve* (Bourhis *et al.*, 2015 ▸), *SHELXT* (Sheldrick, 2015*a* ▸), *SHELXL2018* (Sheldrick, 2015*b* ▸) and *OLEX2* (Dolomanov *et al.*, 2009 ▸).

**Table 2:** Selected bond lengths and com­parisons (Å) of the structures See Fig. 2 ▸ for definitions of distances A–D.

Compound	A	B	C	D	Δ(A–D)	Δ(C—B)
**1**	1.318 (2)	1.320 (2)	1.387 (2)	1.275 (2)	0.043 (4)	0.067 (4)
**1**·HCl (protonated species)	1.299 (3)	1.343 (3)	1.349 (3)	1.294 (2)	0.005 (5)	0.006 (6)
**1**·HCl (neutral species)	1.314 (3)	1.323 (3)	1.393 (3)	1.274 (3)	0.040 (6)	0.070 (6)
**2** (upper)	1.329 (2)	1.316 (2)	1.386 (1)	1.280 (2)	0.049 (4)	0.070 (3)
**2** (lower)	1.325 (2)	1.323 (2)	1.381 (2)	1.289 (2)	0.036 (4)	0.058 (4)
**3**	1.311 (2)	1.333 (1)	1.379 (2)	1.231 (1)	–	0.046 (3)
**4**	1.315 (2)	1.334 (1)	1.366 (2)	1.238 (1)	–	0.032 (3)

**Table 3:** Hydrogen-bond geometry (Å, °) for **1**

*D*—H⋯*A*	*D*—H	H⋯*A*	*D*⋯*A*	*D*—H⋯*A*
N3—H3*A*⋯N2^i^	0.890 (18)	2.061 (18)	2.9414 (15)	169.6 (15)
N3—H3*B*⋯N1^ii^	0.868 (19)	2.083 (19)	2.9238 (16)	162.6 (15)

Symmetry codes: (i) 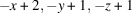 ; (ii) 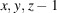 .

**Table 4:** Hydrogen-bond geometry (Å, °) for **1**·HCl

*D*—H⋯*A*	*D*—H	H⋯*A*	*D*⋯*A*	*D*—H⋯*A*
N1—H1*A*⋯N5^i^	0.88	2.12	2.984 (3)	168
N1—H1*B*⋯Cl1^i^	0.88	2.37	3.2432 (18)	173
N3—H3⋯Cl1^ii^	0.79 (3)	2.59 (3)	3.367 (2)	168 (3)
N4—H4*A*⋯O1	0.88	2.07	2.931 (3)	164
N4—H4*B*⋯N3	0.88	1.92	2.795 (3)	171
N6—H6*A*⋯N2^iii^	0.88	2.02	2.903 (3)	177

Symmetry codes: (i) 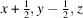 ; (ii) 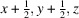 ; (iii) 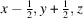 .

**Table 5:** Hydrogen-bond geometry (Å, °) for **2**

*D*—H⋯*A*	*D*—H	H⋯*A*	*D*⋯*A*	*D*—H⋯*A*
O1—H1⋯N3	0.84	1.87	2.7051 (14)	174
O2—H2⋯N6	0.84	1.89	2.7312 (14)	178
N1—H1*A*⋯N5	0.88	2.13	2.9896 (14)	164
N1—H1*B*⋯O1^i^	0.88	1.94	2.8233 (13)	176
N4—H4*A*⋯N2	0.88	2.10	2.9739 (14)	173
N4—H4*B*⋯O2^ii^	0.88	2.00	2.8639 (14)	167
C9—H9*A*⋯O1^iii^	0.99	2.55	3.439 (2)	150

Symmetry codes: (i) 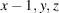 ; (ii) 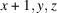 ; (iii) 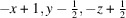 .

**Table 6:** Hydrogen-bond geometry (Å, °) for **3**

*D*—H⋯*A*	*D*—H	H⋯*A*	*D*⋯*A*	*D*—H⋯*A*
N1—H1*A*⋯N2^i^	0.861 (18)	2.099 (19)	2.9454 (16)	167.6 (15)
N1—H1*B*⋯O1^ii^	0.882 (19)	2.01 (2)	2.8832 (15)	170.9 (17)

Symmetry codes: (i) 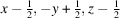 ; (ii) 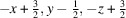 .

**Table 7:** Hydrogen-bond geometry (Å, °) for **4**

*D*—H⋯*A*	*D*—H	H⋯*A*	*D*⋯*A*	*D*—H⋯*A*
N2—H2*A*⋯N1^i^	0.89 (2)	2.07 (2)	2.9550 (15)	178 (1)
N2—H2*B*⋯O1^ii^	0.90 (2)	1.97 (2)	2.8588 (14)	170 (1)

Symmetry codes: (i) 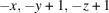 ; (ii) 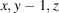 .

## References

[bb1] Beach, S. A., Rheingold, A. L. & Doerrer, L. H. (2021). *Polyhedron*, **208**, 115403.

[bb2] Berry, J. F., Cotton, F. A., Daniels, L. M., Murillo, C. A. & Wang, X. (2003). *Inorg. Chem.* **42**, 2418–2427. 10.1021/ic0262740 12665379

[bb3] Bordwell, F. G. (1988). *Acc. Chem. Res.* **21**, 456–463.

[bb4] Bordwell, F. G. & Ji, G. Z. (1991). *J. Am. Chem. Soc.* **113**, 8398–8401.

[bb5] Bourhis, L. J., Dolomanov, O. V., Gildea, R. J., Howard, J. A. K. & Puschmann, H. (2015). *Acta Cryst.* A**71**, 59–75. 10.1107/S2053273314022207 PMC4283469 25537389

[bb6] Brogden, D. W. & Berry, J. F. (2016). *Comments Inorg. Chem.* **36**, 17–37.

[bb7] Bruker (2016). *APEX3*, *SAINT-Plus*, and *SADABS*. Bruker AXS Inc., Madison, Wisconsin, USA.

[bb8] Bruker (2017). *APEX3* and *SAINT-Plus*. Bruker AXS Inc., Madison, Wisconsin, USA.

[bb9] Chang, W.-C., Chang, C.-W., Sigrist, M., Hua, S.-A., Liu, T.-J., Lee, G.-H., Jin, B.-Y., Chen, C. & Peng, S. (2017). *Chem. Commun.* **53**, 8886–8889. 10.1039/c7cc05449a 28737805

[bb10] Chipman, J. A. & Berry, J. F. (2018*a*). *Chem. Eur. J.* **24**, 1494–1499. 10.1002/chem.201704588 29124828

[bb11] Chipman, J. A. & Berry, J. F. (2018*b*). *Inorg. Chem.* **57**, 9354–9363. 10.1021/acs.inorgchem.8b01331 30024159

[bb12] Chipman, J. A. & Berry, J. F. (2020). *Chem. Rev.* **120**, 2409–2447. 10.1021/acs.chemrev.9b00540 32045223

[bb13] Ding, D.-D., Xu, X., Wu, Z.-W., Zhou, W.-H., Chen, R. & Xu, Z.-G. (2015). *Acta Phys.-Chim. Sin.* **31**, 1323–1330.

[bb14] Dolomanov, O. V., Bourhis, L. J., Gildea, R. J., Howard, J. A. K. & Puschmann, H. (2009). *J. Appl. Cryst.* **42**, 339–341.

[bb15] Elvidge, J. A. & Linstead, R. P. (1954). *J. Chem. Soc.* pp. 442–448.

[bb16] Elvidge, J. A., Linstead, R. P. & Salaman, A. M. (1959). *J. Chem. Soc.* pp. 208–215.

[bb17] Emmeluth, C., Suhm, M. A. & Luckhaus, D. (2003). *J. Chem. Phys.* **118**, 2242–2255.

[bb18] Frisch, M. J., Trucks, G. W., Schlegel, H. B., Scuseria, G. E., Robb, M. A., Cheeseman, J. R., Scalmani, G., Barone, V., Petersson, G. A., Nakatsuji, H., Li, X., Caricato, M., Marenich, A. V., Bloino, J., Janesko, B. G., Gomperts, R., Mennucci, B., Hratchian, H. P., Ortiz, J. V., Izmaylov, A. F., Sonnenberg, J. L., Williams, Ding, F., Lipparini, F., Egidi, F., Goings, J., Peng, B., Petrone, A., Henderson, T., Ranasinghe, D., Zakrzewski, V. G., Gao, J., Rega, N., Zheng, G., Liang, W., Hada, M., Ehara, M., Toyota, K., Fukuda, R., Hasegawa, J., Ishida, M., Nakajima, T., Honda, Y., Kitao, O., Nakai, H., Vreven, T., Throssell, K., Montgomery Jr., J. A., Peralta, J. E., Ogliaro, F., Bearpark, M. J., Heyd, J. J., Brothers, E. N., Kudin, K. N., Staroverov, V. N., Keith, T. A., Kobayashi, R., Normand, J., Raghavachari, K., Rendell, A. P., Burant, J. C., Iyengar, S. S., Tomasi, J., Cossi, M., Millam, J. M., Klene, M., Adamo, C., Cammi, R., Ochterski, J. W., Martin, R. L., Morokuma, K., Farkas, O., Foresman, J. B. & Fox, D. J. (2016). *GAUSSIAN16*. Revision C.01. Gaussian Inc., Wallingford, CT, USA. https://gaussian.com/.

[bb19] Godsi, O., Turner, B., Suwinska, K., Peskin, U. & Eichen, Y. (2004). *J. Am. Chem. Soc.* **126**, 13519–13525. 10.1021/ja046311h 15479108

[bb20] Hsiao, C.-J., Lai, S.-H., Chen, I.-C., Wang, W.-Z. & Peng, S.-M. (2008). *J. Phys. Chem. A*, **112**, 13528–13534. 10.1021/jp8081326 19053565

[bb21] Jasinski, J. P., Butcher, R. J., Yathirajan, H. S., Narayana, B., Mallesha, L. & Mohana, K. N. (2009). *J. Chem. Crystallogr.* **39**, 453–457.

[bb22] Kerru, N., Gummidi, L., Maddila, S., Gangu, K. K. & Jonnalagadda, S. B. (2020). *Molecules*, **25**, 1909. 10.3390/molecules25081909 PMC7221918 32326131

[bb23] Kolbe, A., Plass, M., Kresse, H., Kolbe, A., Drabowicz, J. & Zurawinski, R. (1997). *J. Mol. Struct.* **436–437**, 161–166.

[bb24] Krause, L., Herbst-Irmer, R., Sheldrick, G. M. & Stalke, D. (2015). *J. Appl. Cryst.* **48**, 3–10. 10.1107/S1600576714022985 PMC4453166 26089746

[bb25] Lescouëzec, R., Marinescu, G., Carmen Muñoz, M., Luneau, D., Andruh, M., Lloret, F., Faus, J., Julve, M., Antonio Mata, J., Llusar, R. & Cano, J. (2001). *New J. Chem.* **25**, 1224–1235.

[bb26] Liu, I. P.-C., Chen, C.-H., Chen, C.-F., Lee, G.-H. & Peng, S.-M. (2009). *Chem. Commun.* pp. 577–579. 10.1039/b817032k 19283296

[bb27] Liu, I. P.-C., Wang, W.-Z. & Peng, S.-M. (2009). *Chem. Commun.* pp. 4323–4331. 10.1039/b904719k 19597588

[bb28] March, J. (1992). In *Advanced Organic Chemistry: Reactions, Mechanisms, and Structure*, 4th ed. New York: Wiley.

[bb29] Mason, R. (1961). *Acta Cryst.* **14**, 720–724.

[bb30] Pinner, A. (1883). *Ber. Dtsch Chem. Ges.* **16**, 352–363.

[bb31] Roy, M. D., Trenerry, M. J., Thakuri, B., Macmillan, S. N., Liptak, M. D., Lancaster, K. M. & Berry, J. F. (2022). *Inorg. Chem.* **61**, 3443–3457. 10.1021/acs.inorgchem.1c03346 35175754

[bb32] Sheldrick, G. M. (2015*a*). *Acta Cryst.* A**71**, 3–8.

[bb33] Sheldrick, G. M. (2015*b*). *Acta Cryst.* C**71**, 3–8.

[bb34] Tsai, C.-S., Liu, I. P.-C., Tien, F.-W., Lee, G.-H., Yeh, C.-Y., Chen, C. & Peng, S. (2013). *Inorg. Chem. Commun.* **38**, 152–155.

[bb35] Yu, M., Huang, X. & Gao, F. (2012). *Acta Cryst.* E**68**, o2738. 10.1107/S1600536812035672 PMC3435750 22969621

