# Supplementary material for: Clarifying the structures of imidines: using crystallographic characterization to identify tautomers and localized systems of π-bonding
Source: Acta Crystallogr C Struct Chem. 2023 Mar 15;79(Pt 4):133–41. doi: 10.1107/S2053229623002036 (PMC10074040; doi:10.1107/S2053229623002036)
Supplement: Supplementary file 12 [file c-79-00133-sup12.pdf]

STRUCTURAL CHEMISTRY

Volume 79 (2023)

Supporting information for article:

Clarifying the structures of imidines: using crystallographic characterization to identify tautomers and localized systems of π-bonding

Michael M. Aristov, Han Geng, James W. Harris and John F. Berry

Table of Contents:

IR Data 2-5

MS Data 6-9

NMR Data 10-15

Computational Data 16

Supplemental Crystallographic Images 17-25

Reference 25

1

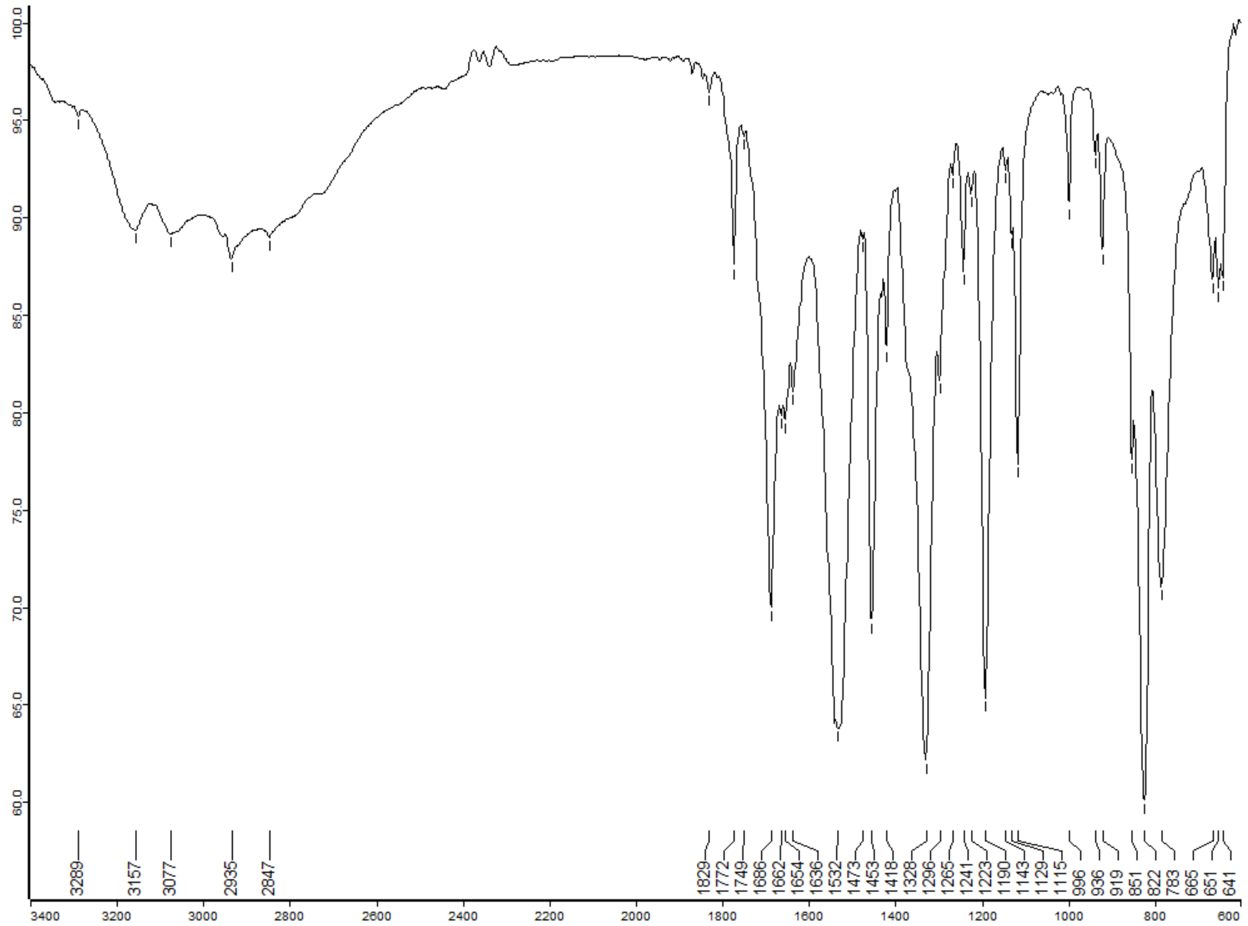
100.0
95.0
90.0
85.0
80.0
75.0
70.0
65.0
60.0
3289
3157
3077
2935
2847
1829
1772
1749
1686
1662
1654
1636
1532
1473
1453
1418
1328
1296
1265
1241
1223
1190
1143
1129
1115
996
936
919
851
822
783
665
651
641
3400
3200
3000
2800
2600
2400
2200
2000
1800
1600
1400
1200
1000
800
600

2

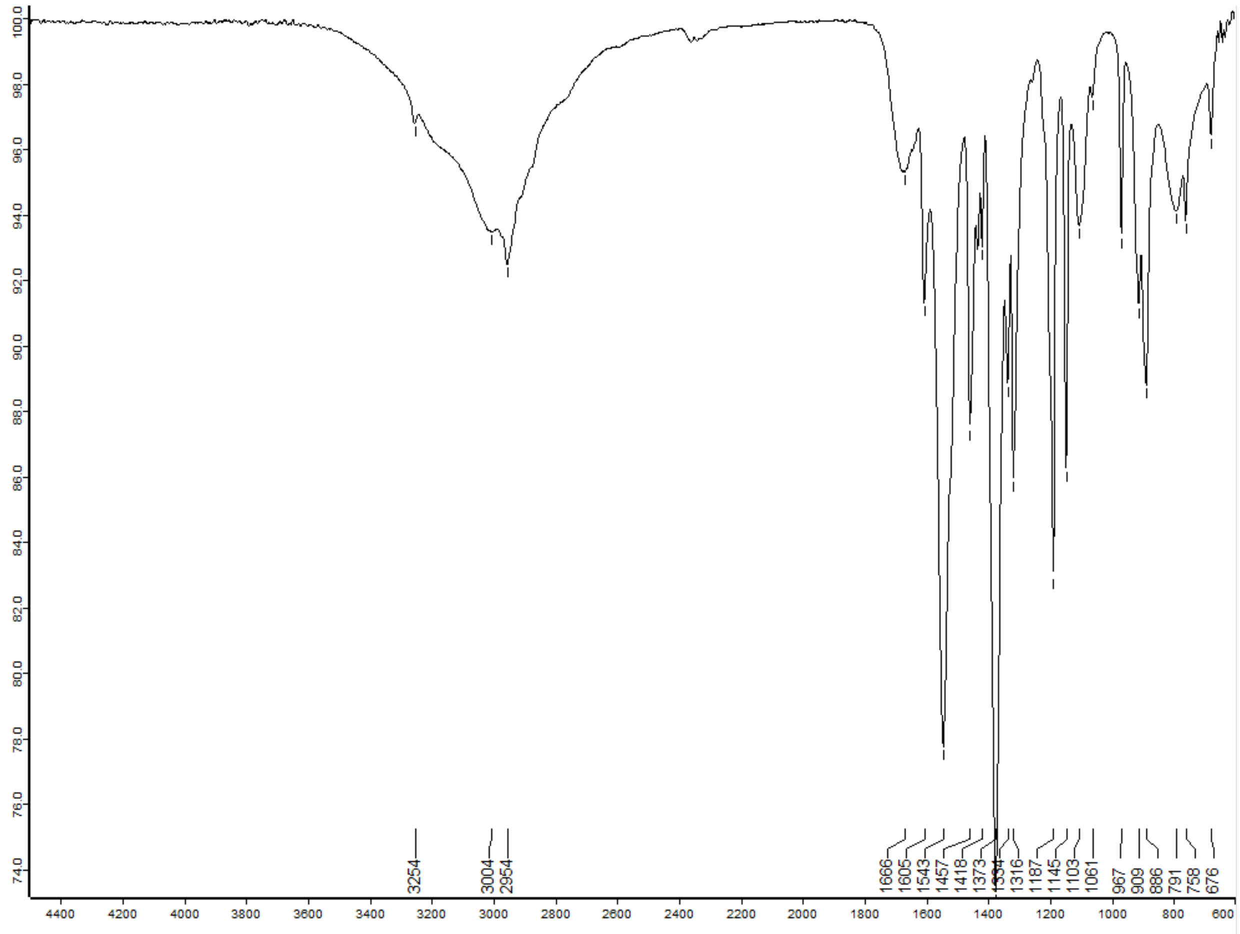
100.0
98.0
96.0
94.0
92.0
90.0
88.0
86.0
84.0
82.0
80.0
78.0
76.0
74.0
4400
4200
4000
3800
3600
3400
3200
3000
2800
2600
2400
2200
2000
1800
1600
1400
1200
1000
800
600
3254
3004
2954
1666
1605
1543
1457
1418
1373
1316
1187
1145
1103
1061
967
909
886
791
758
676

3

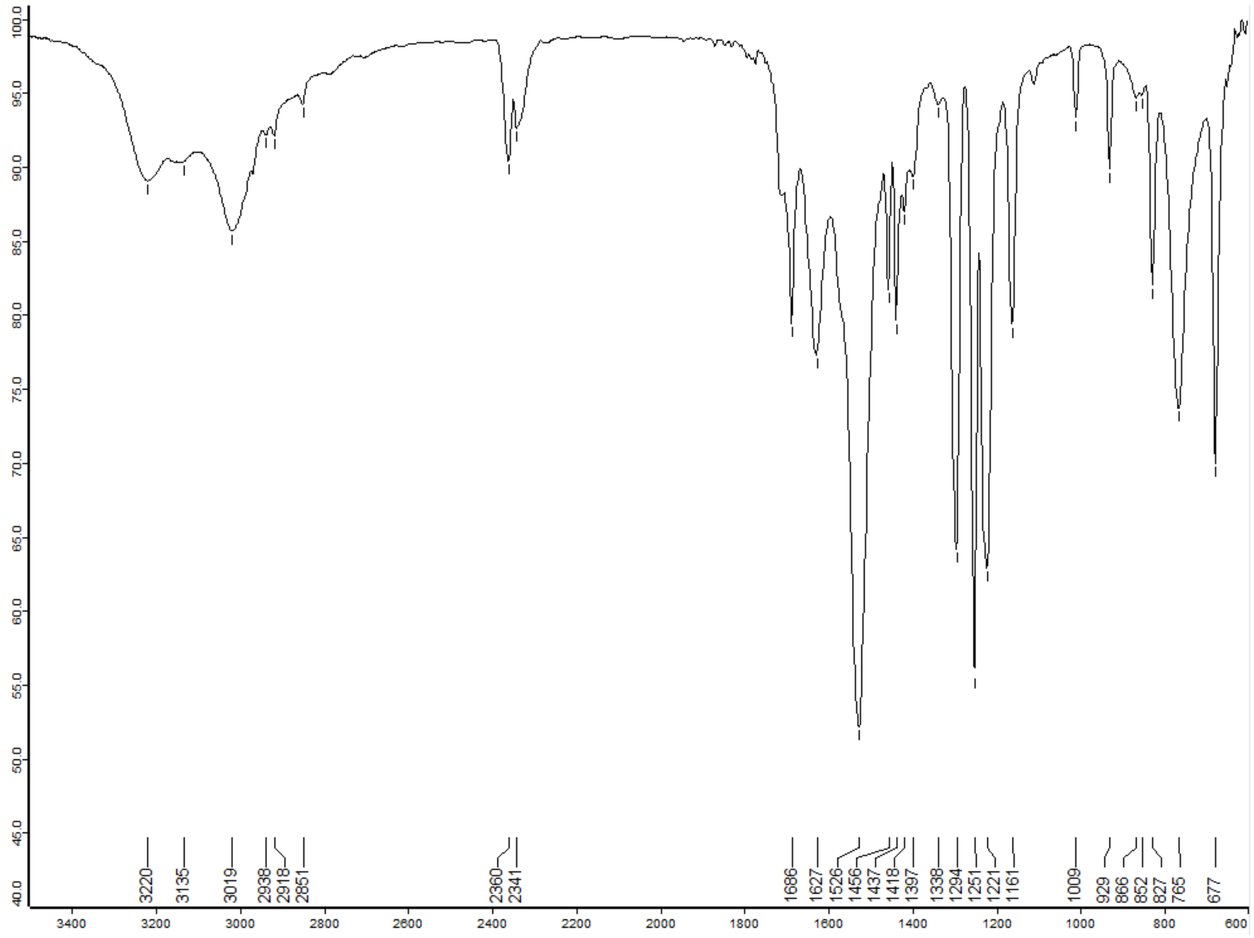
100.0
95.0
90.0
85.0
80.0
75.0
70.0
65.0
60.0
55.0
50.0
45.0
40.0
3220
3135
3019
2938
2918
2851
2360
2341
1686
1627
1526
1456
1437
1418
1397
1338
1294
1251
1221
1161
1009
929
866
852
827
765
677
3400
3200
3000
2800
2600
2400
2200
2000
1800
1600
1400
1200
1000
800
600

4

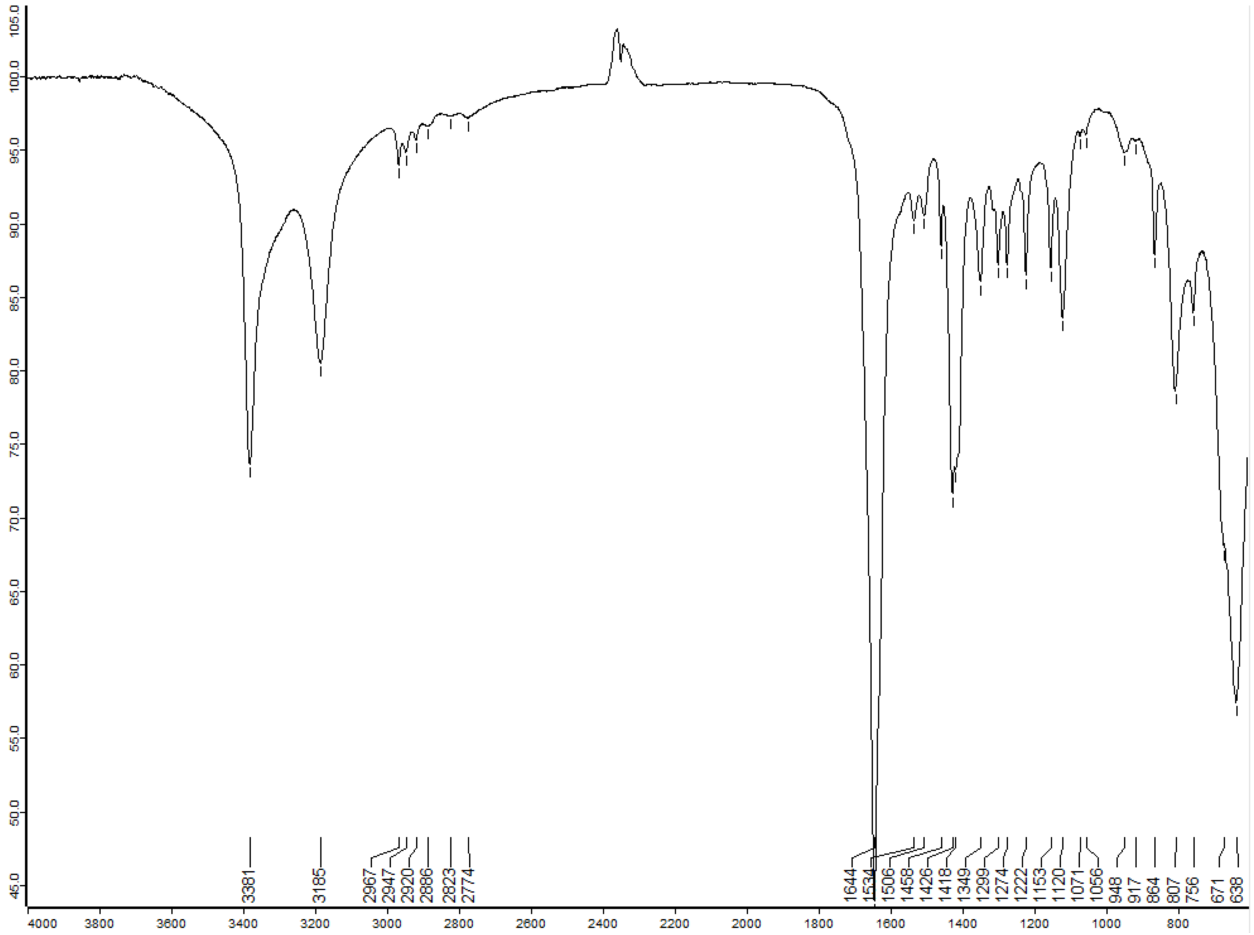
105.0
100.0
95.0
90.0
85.0
80.0
75.0
70.0
65.0
60.0
55.0
50.0
45.0
3381
3185
2967
2947
2920
2886
2823
2774
1644
1534
1506
1458
1426
1418
1349
1299
1274
1222
1153
1120
1071
1056
948
917
864
807
756
671
638
4000
3800
3600
3400
3200
3000
2800
2600
2400
2200
2000
1800
1600
1400
1200
1000
800

Mass Spec Data:

1

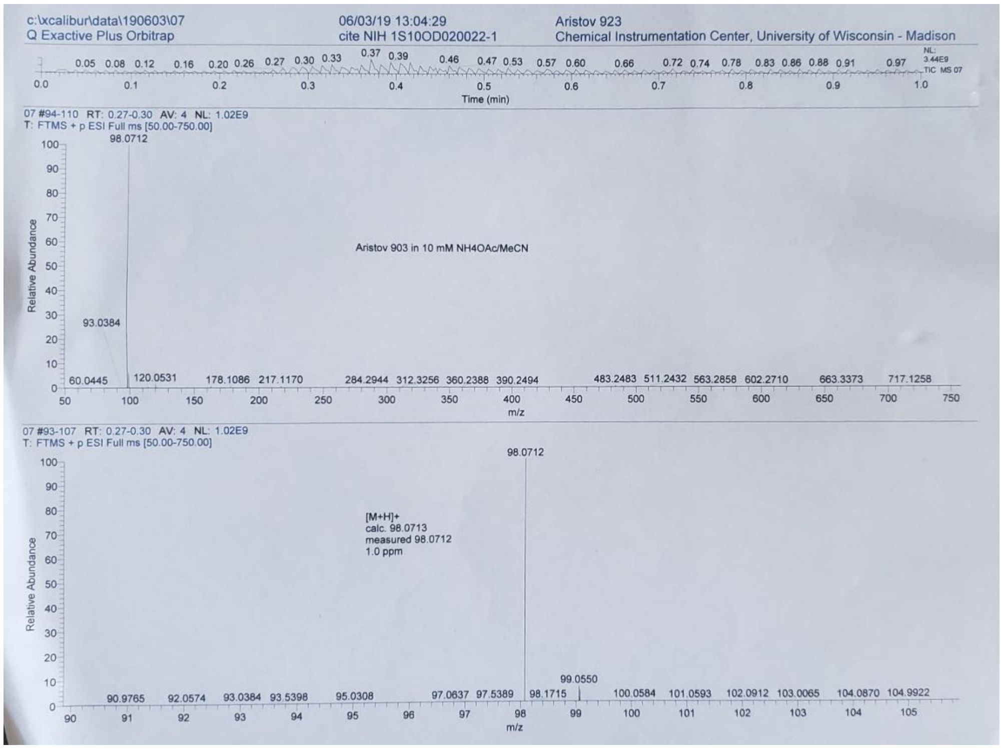

3

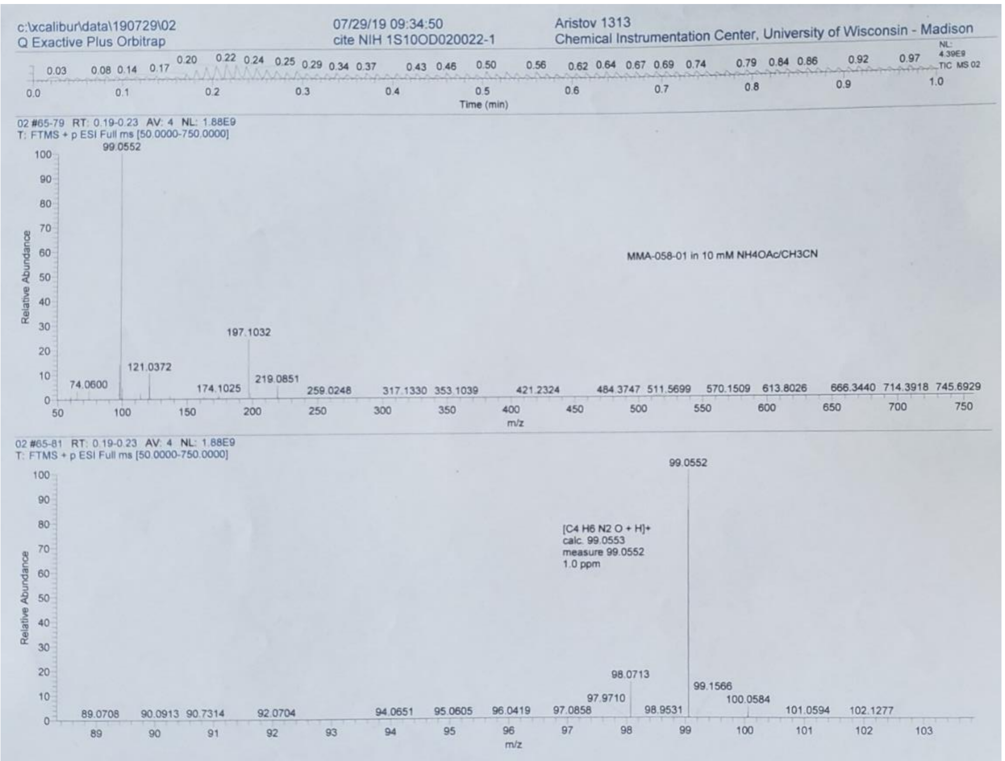

ESI (*m*/*z*): ($[C_8H_{10}N_4O](H_2O)+H]^+$) 197.1032

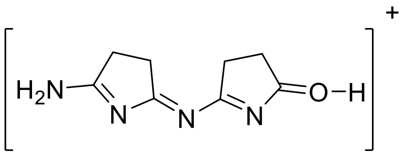

$+H_2O$

ESI (*m*/*z*): ($[HNNO5+Na]^+$) 121.0372

2

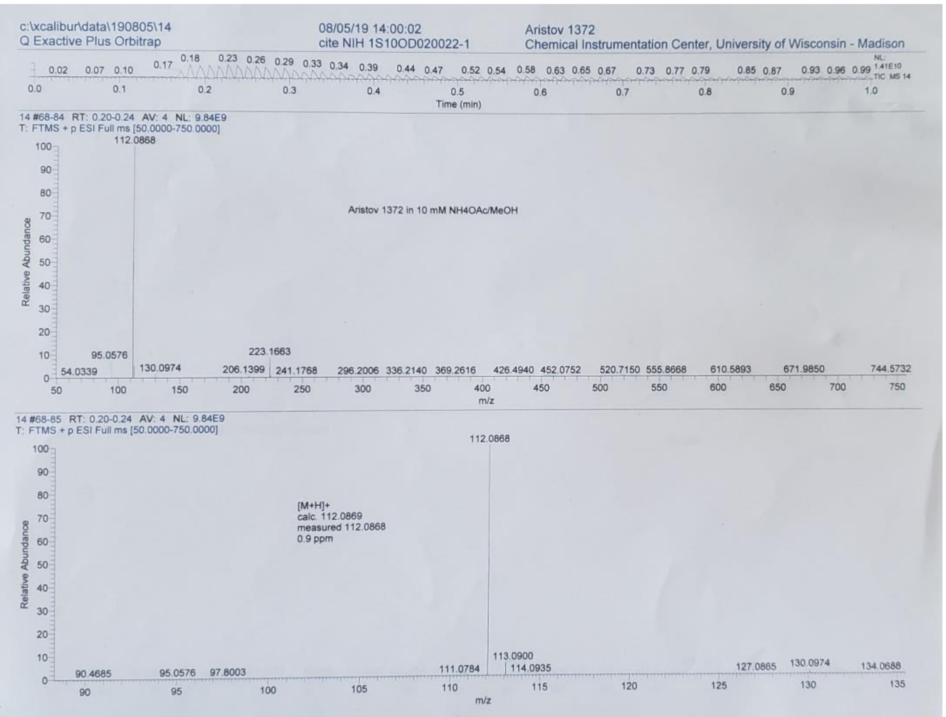

ESI (*m*/*z*): ($[C_{10}H_{15}N_5]$+$NH_4]^+$) 223.17

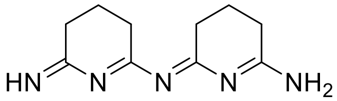

+NH_4

4

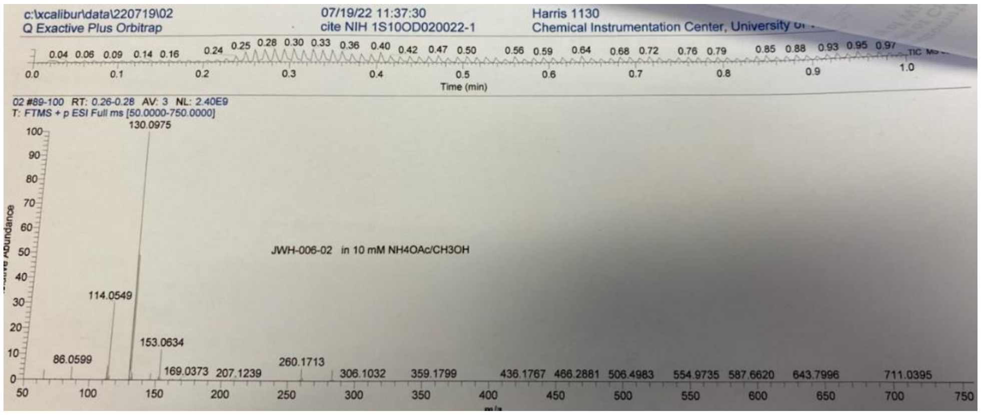

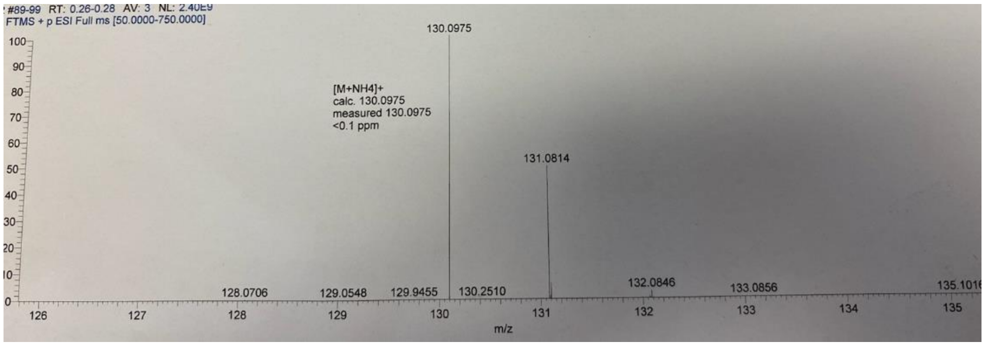

ESI (*m*/*z*): ([HONO6]+NH_4]$^+$) 131.0814

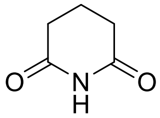

+NH_4

NMR Data:

1: ^{1}H NMR (400 MHz, **1** in DMSO) δ 7.37 (s, 3H), 2.46 (s, 4H).

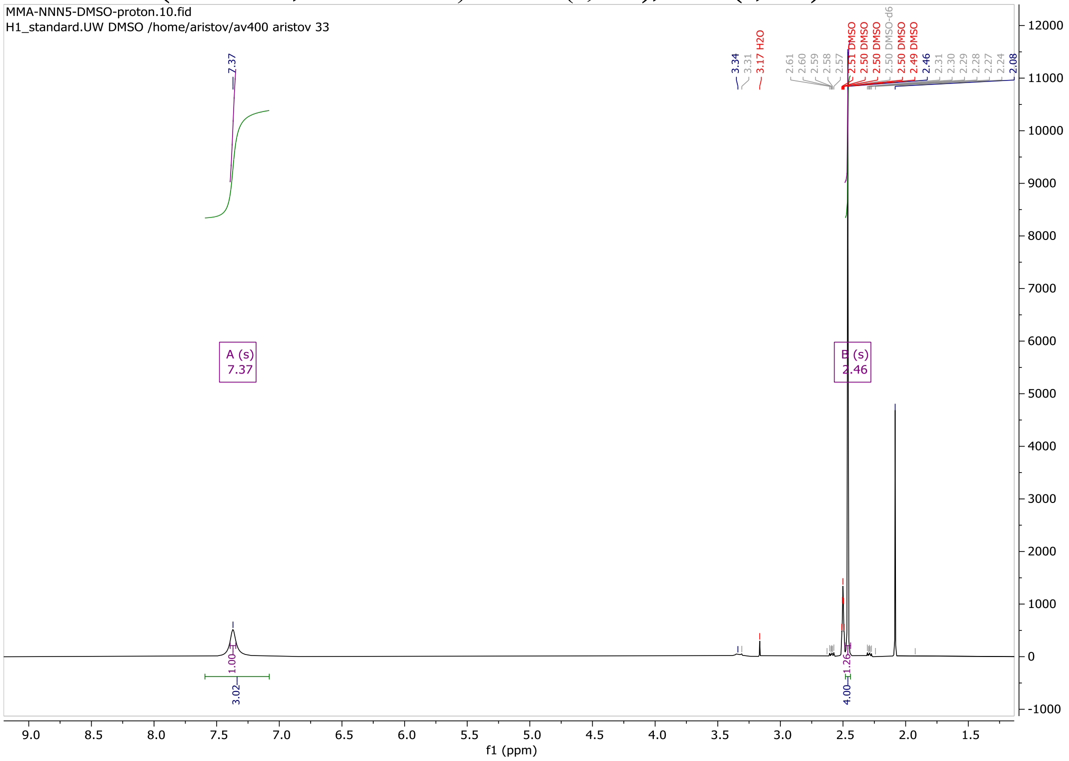

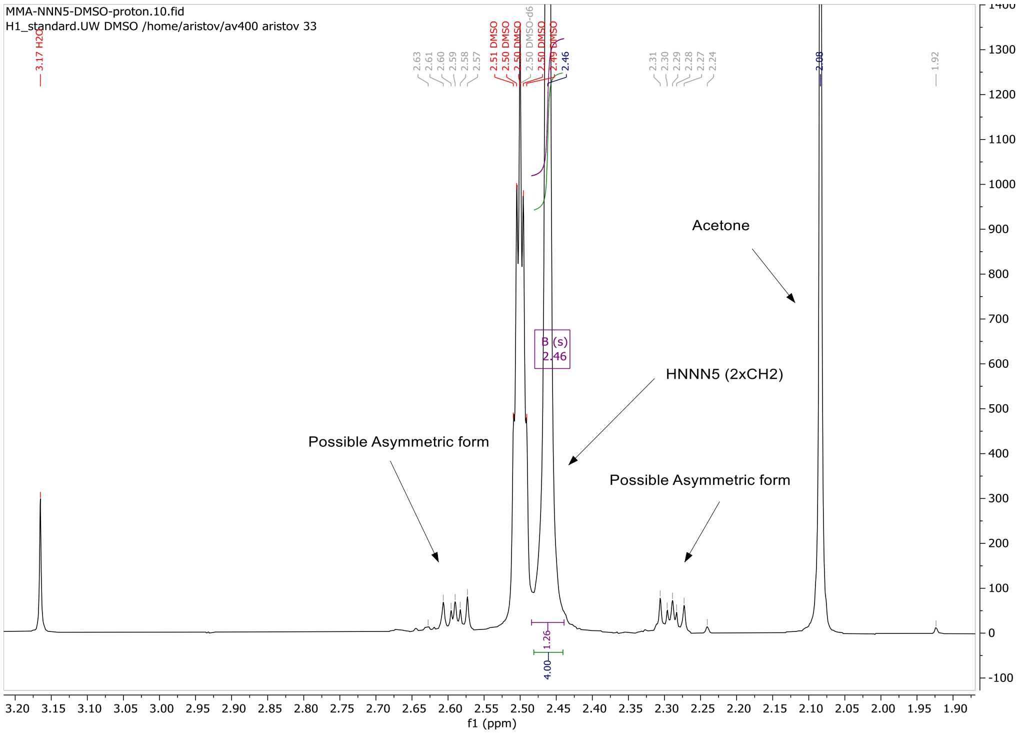

3: ^{1}H NMR (400 MHz, **3** DMSO) δ 8.30 (s, 1H), 8.07 (s, 1H), 2.67 – 2.56 (m, 2H), 2.34 – 2.25 (m, 2H).

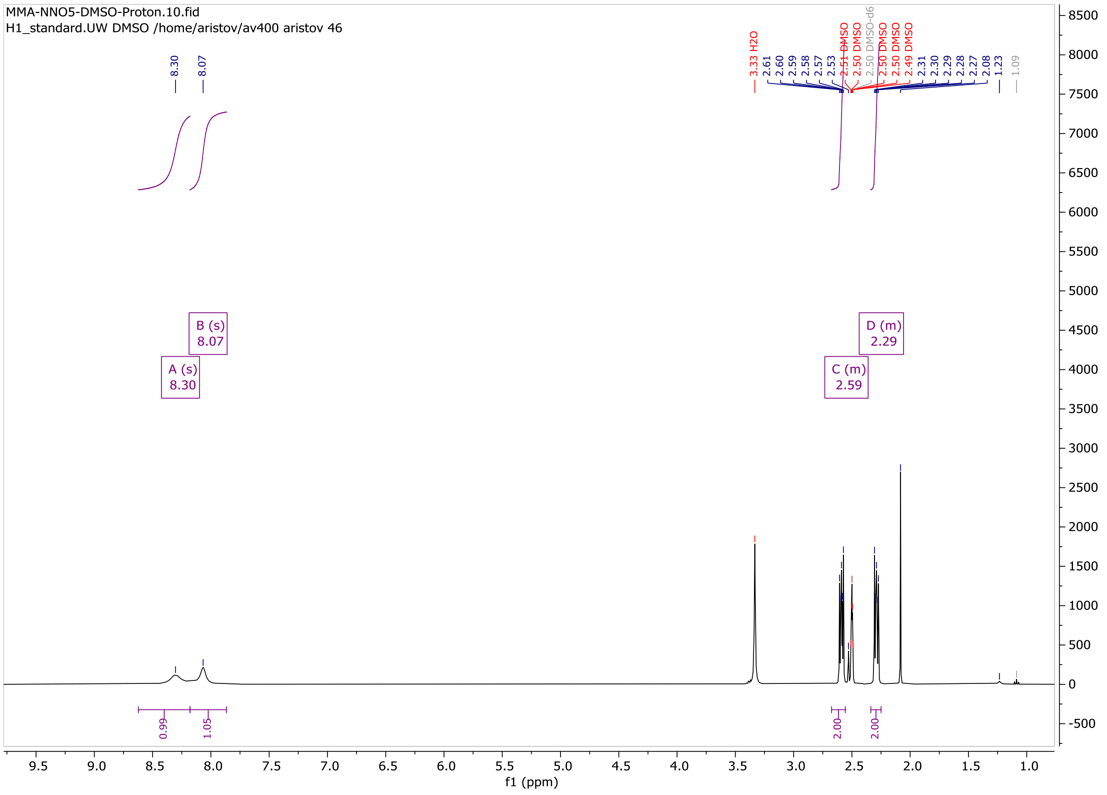

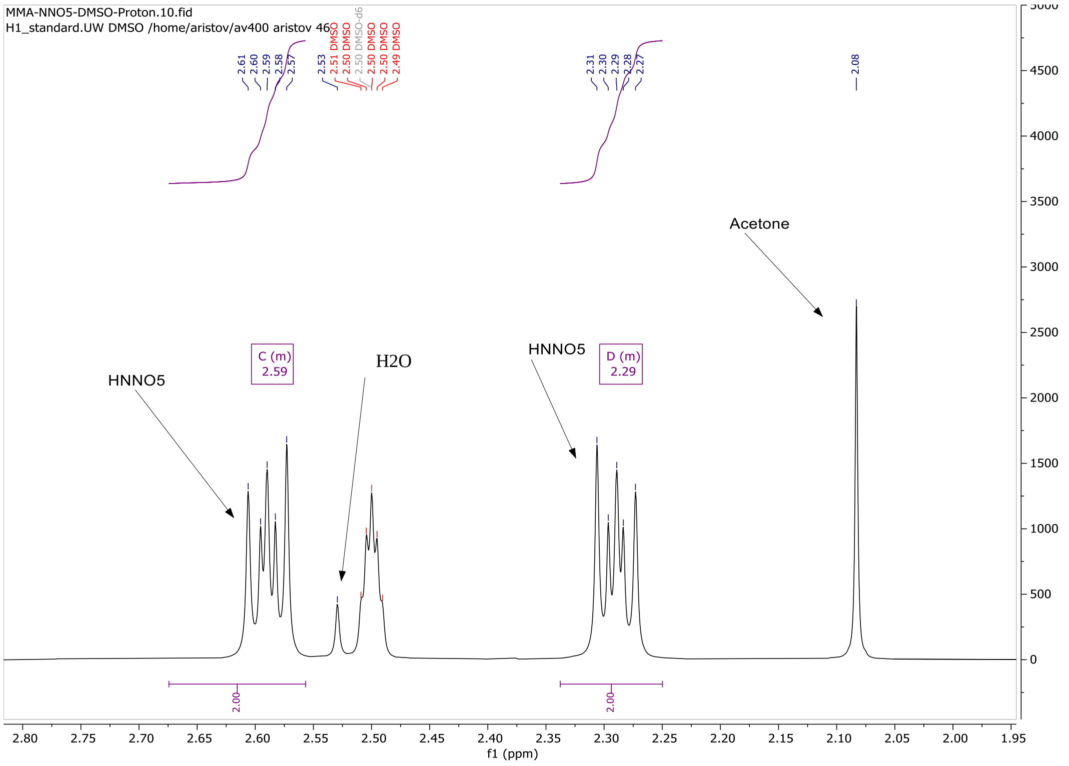

3: ^{13}C NMR (101 MHz, **3** in DMSO) δ 193.51, 31.33, 30.72, 28.08.

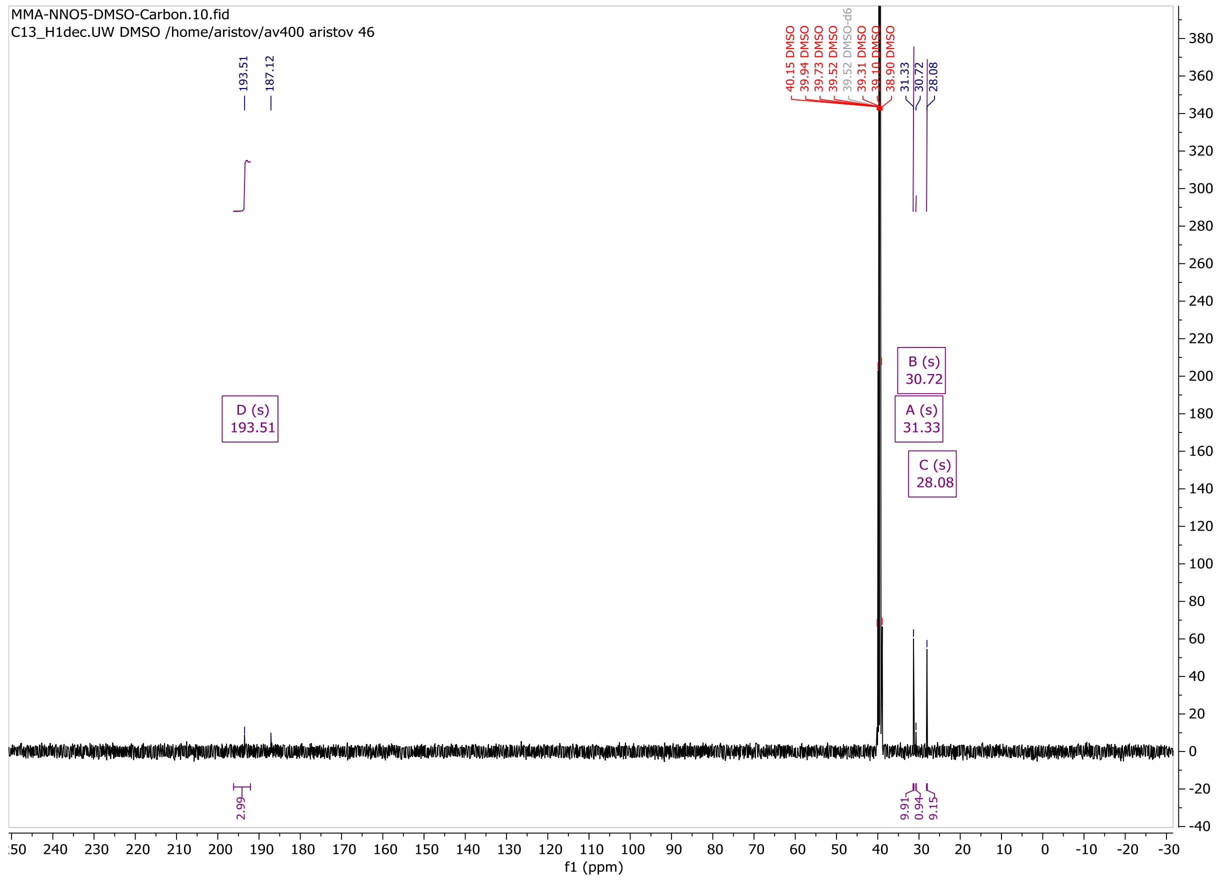

2: ^{1}H NMR (400 MHz, **2** in DMSO) δ 7.05 (s, 3H), 2.20 (t, *J* = 6.5 Hz, 4H), 1.80 – 1.57 (q, 2H).

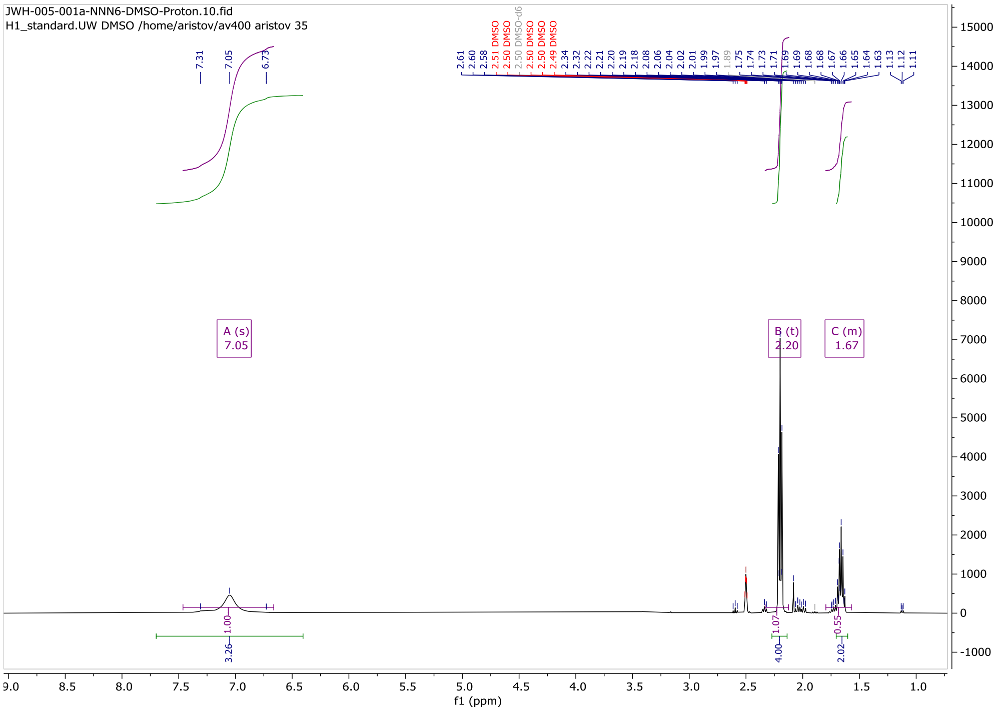

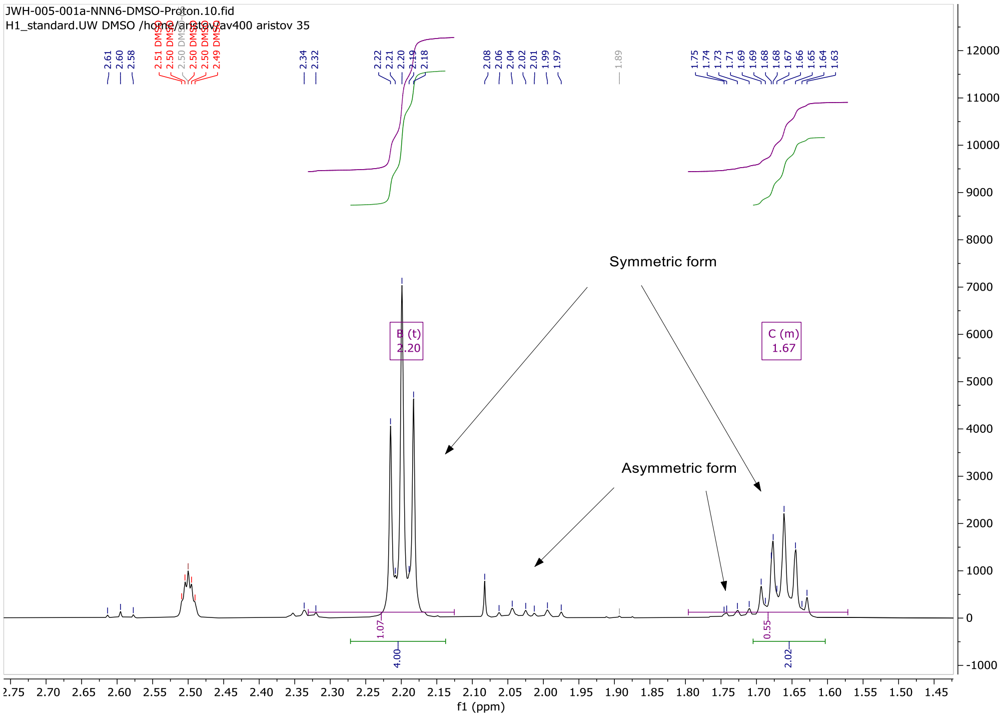

2: ^{13}C NMR (101 MHz, **2** in DMSO) δ 168.09, 27.53, 19.45.

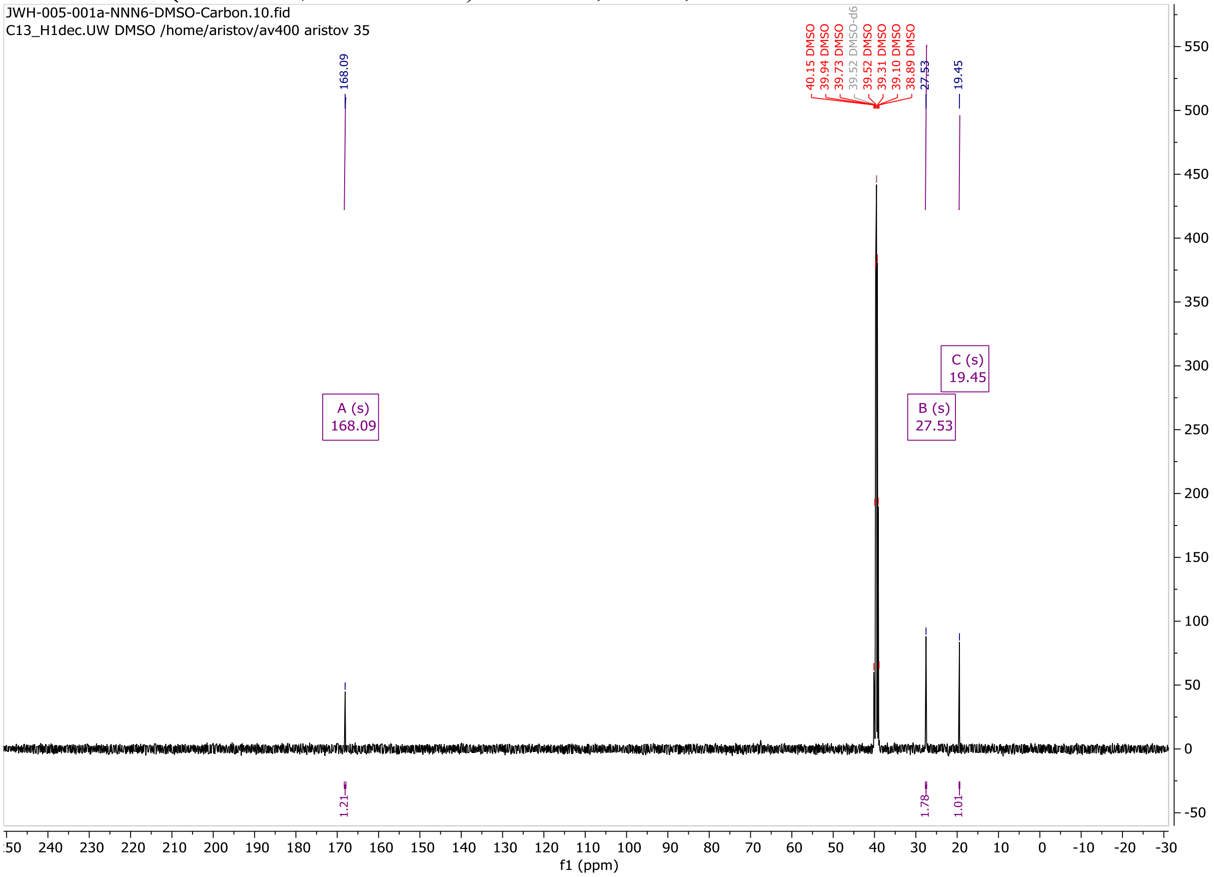

4: ^{1}H NMR (400 MHz, **4** DMSO-d_6) δ, 7.35 (s, 1H), 6.80 (s, 1H), 2.24 (t, J = 7.7 Hz, 2H), 1.88 (t, J = 7.3 Hz, 2H), 1.78 (p, J = 7.4 Hz, 2H). The remaining peaks are either unreacted starting material, **3**, or glutarimide.

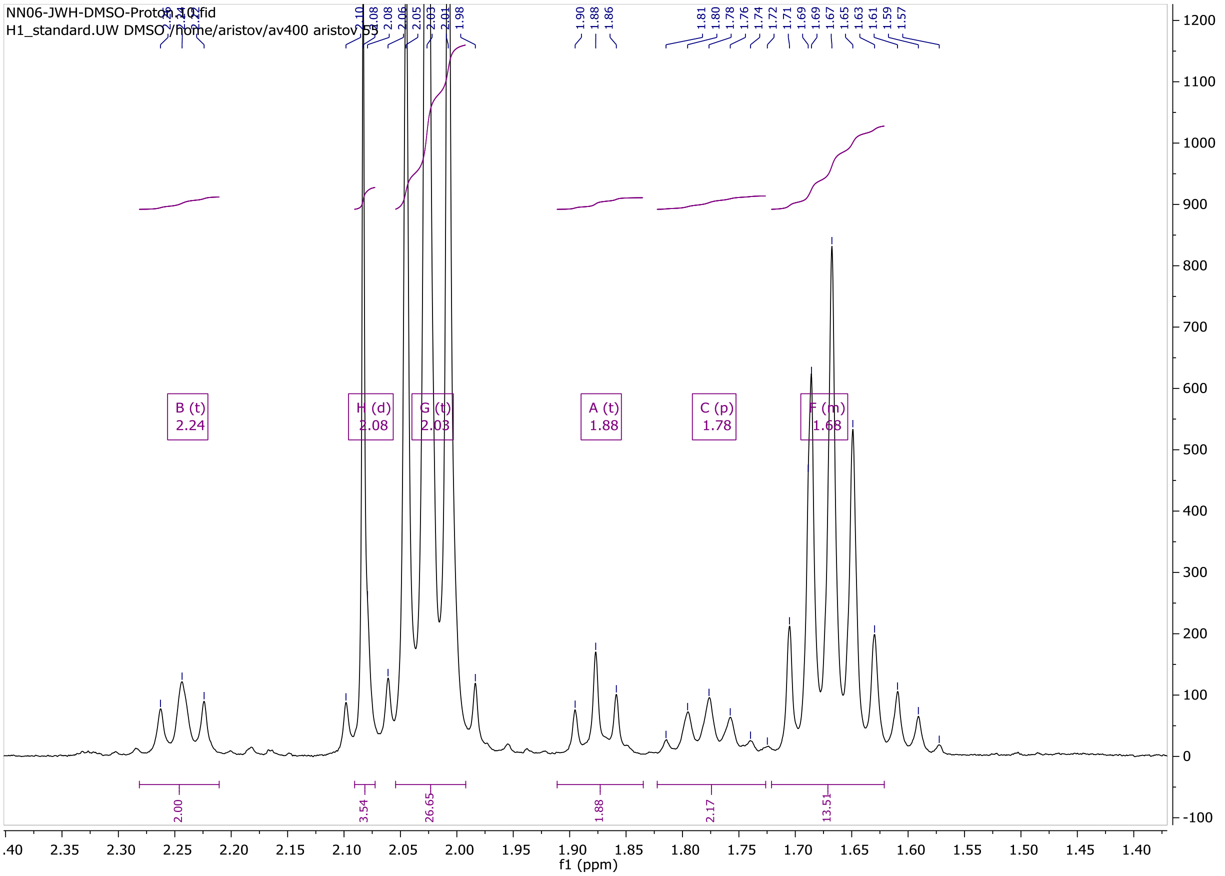

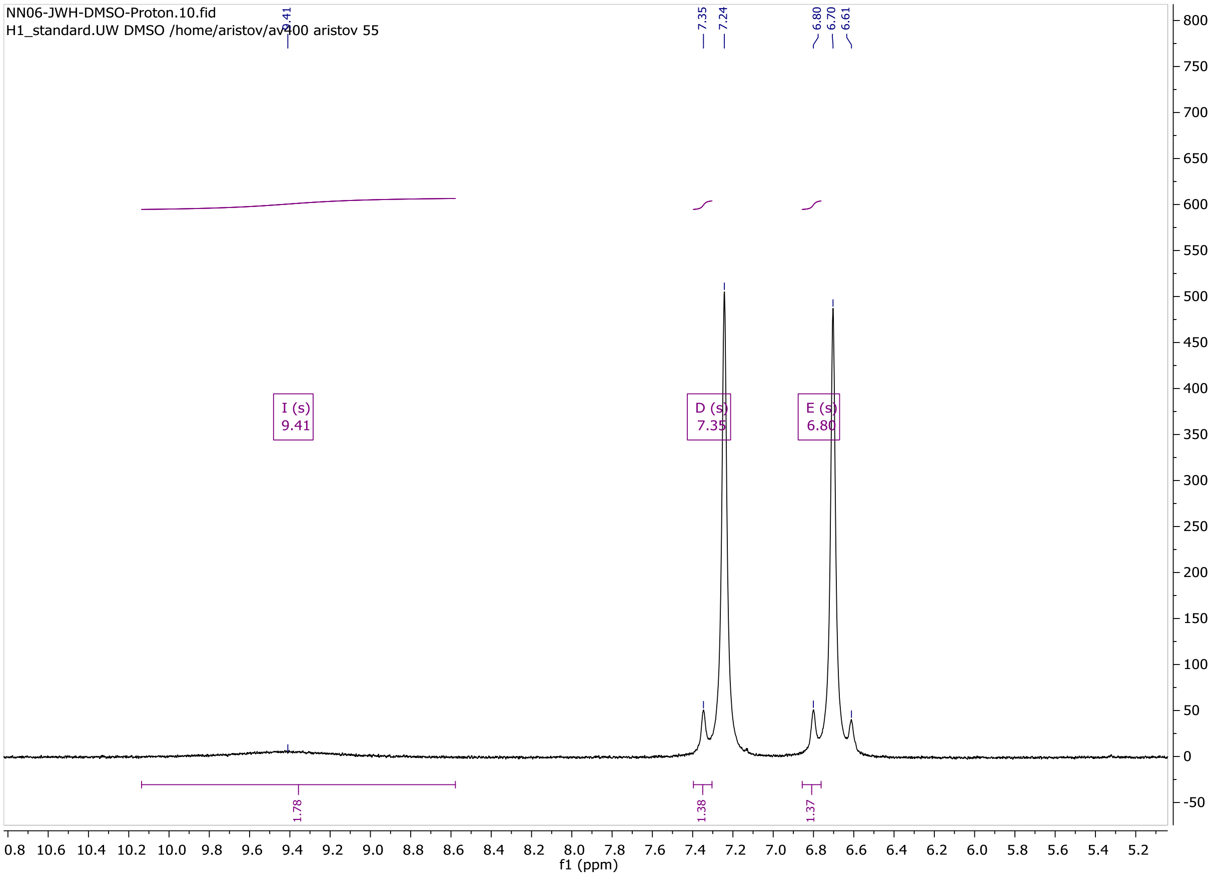
NN06-JWH-DMSO-Proton.10.fid
H1_standard.UW DMSO /home/aristov/av400 aristov 55
9.41
7.35
7.24
6.80
6.70
6.61
I (s)
9.41
D (s)
7.35
E (s)
6.80
1.78
1.38
1.37
f1 (ppm)

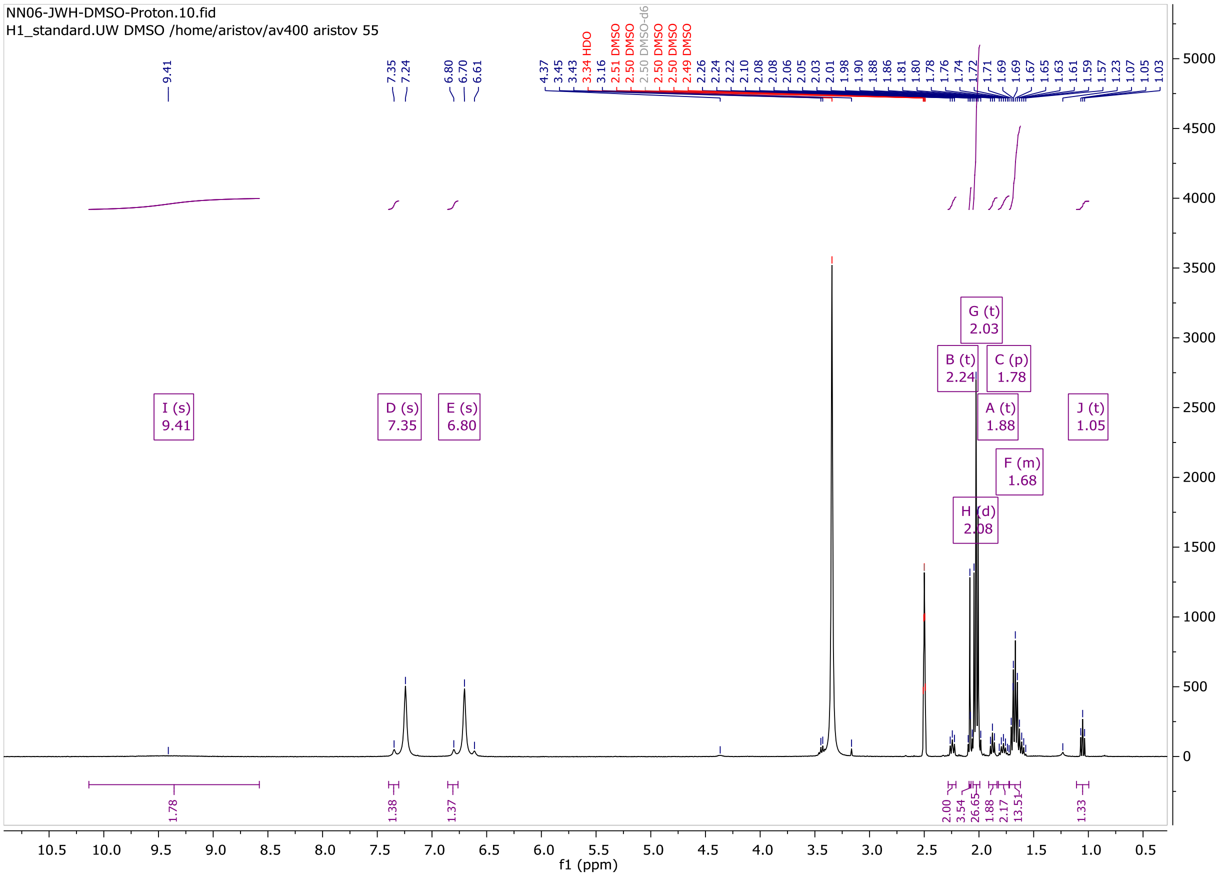
NN06-JWH-DMSO-Proton.10.fid
H1_standard.UW DMSO /home/aristov/av400 aristov 55
I (s) 9.41
D (s) 7.35
E (s) 6.80
G (t) 2.03
B (t) 2.24
C (p) 1.78
A (t) 1.88
J (t) 1.05
F (m) 1.68
H (d) 2.08
f1 (ppm)

Calculations:

Table S1. XYZ coordinates from the optimized asymmetric structure of **1**.

Atom	X	Y	Z
C	0.649857	1.277596	-0.00554
C	-0.87957	1.208895	0.007742
C	-1.165	-0.29754	-0.00051
N	0.036009	-1.01282	-0.00076
C	1.005749	-0.19268	0.001706
N	2.298247	-0.57871	0.049523
H	2.487487	-1.54654	-0.09035
H	3.013518	0.059235	-0.21267
N	-2.28135	-0.86433	-0.00814
H	-3.02497	-0.18848	-0.00903
H	-1.30665	1.666701	0.89213
H	-1.32716	1.683164	-0.85749
H	1.050836	1.764418	-0.88898
H	1.070304	1.774916	0.861671

Table S2. XYZ coordinates from the optimized symmetric structure of **1**.

Atom	X	Y	Z
C	0.738432	1.272389	0.093736
C	-0.78701	1.239713	-0.0961
C	-1.1574	-0.23124	-0.00404
N	0.025415	-0.93835	0.000991
C	1.175065	-0.17634	0.005833
N	2.371288	-0.54612	-0.03507
H	2.465645	-1.54601	-0.07787
H	0.017593	-1.93459	0.010076
N	-2.27434	-0.79566	0.041777
H	-3.02215	-0.12755	0.023393
H	-1.32447	1.814511	0.647444
H	-1.08008	1.609986	-1.07252
H	1.255905	1.862327	-0.64936
H	1.016463	1.655097	1.068347

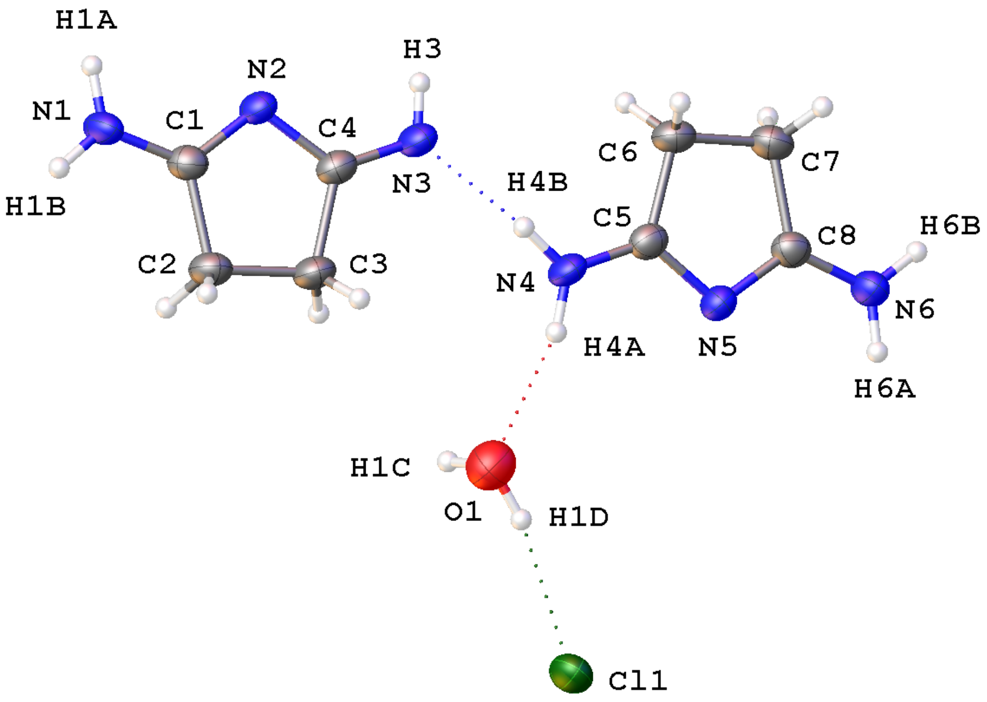

Figure S1. A molecular drawing of the structure of **1**•HCl shown with 50% probability ellipsoids.

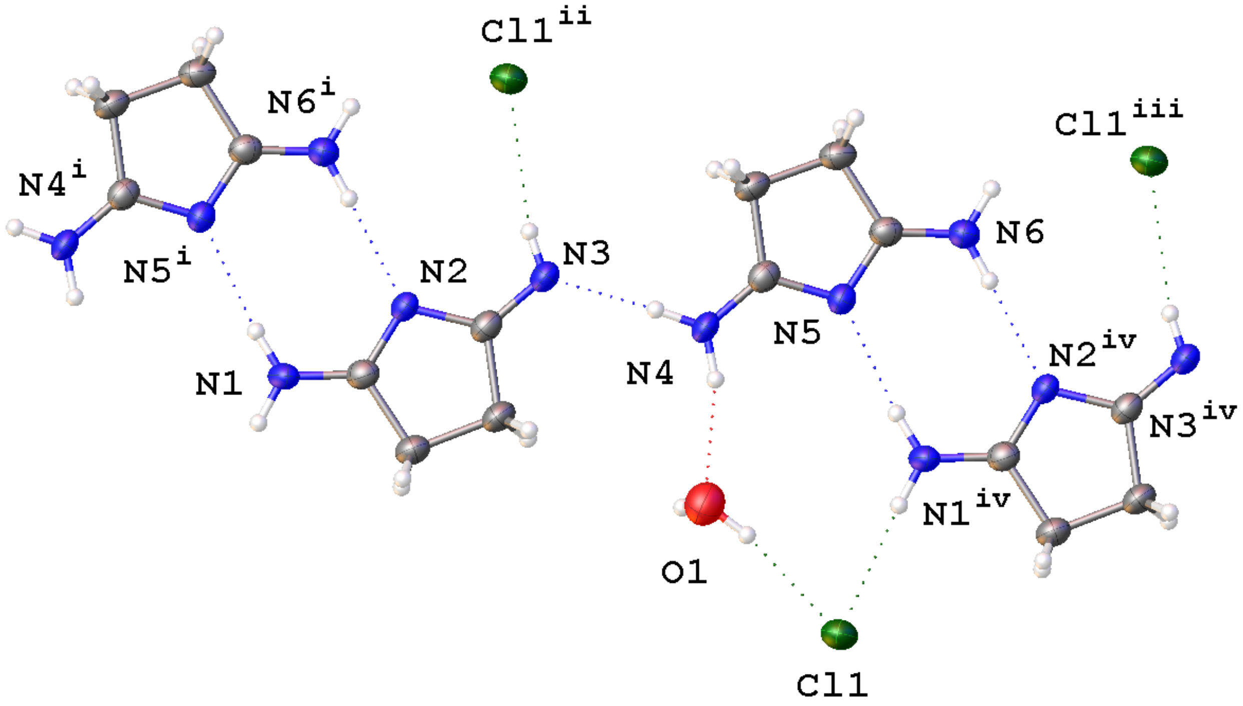

Figure S2. A molecular drawing of **1**•HCl shown with 50% probability ellipsoids, with emphasis placed on the layered hydrogen bonding system. [Symmetry code: i: 1/2+X,-1/2+Y,+Z; ii: 1/2+X,1/2+Y,+Z ; iii: -1/2+X,1/2+Y,+Z ; iv: +X,1+Y,+Z.]

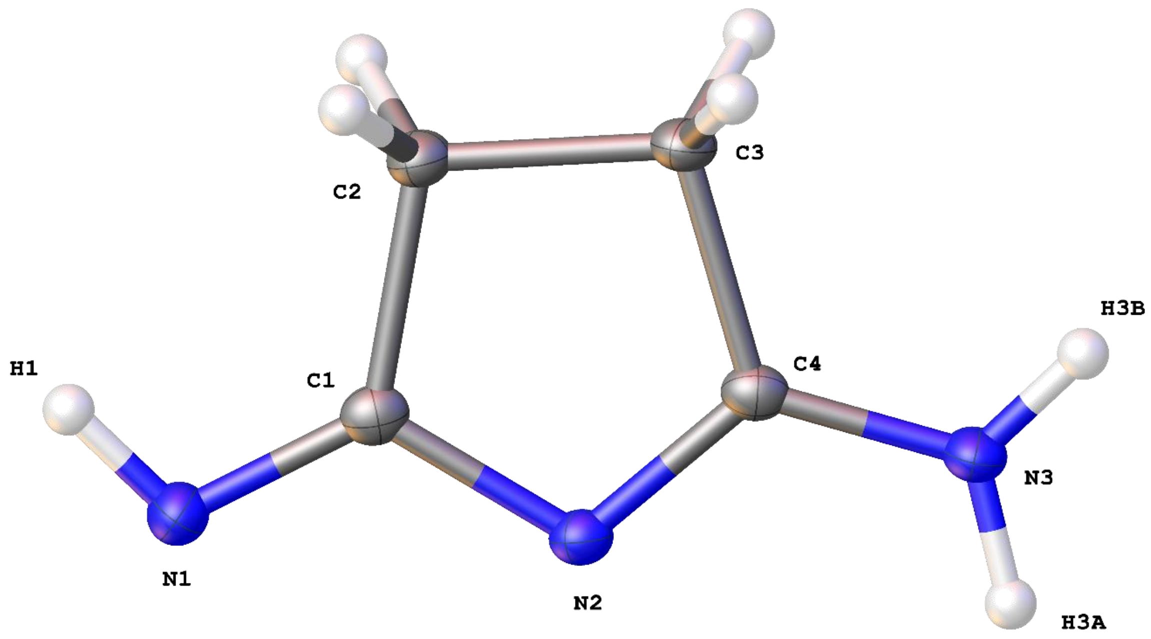

Figure S3. A molecular drawing of **1** shown with 50% probability ellipsoids.

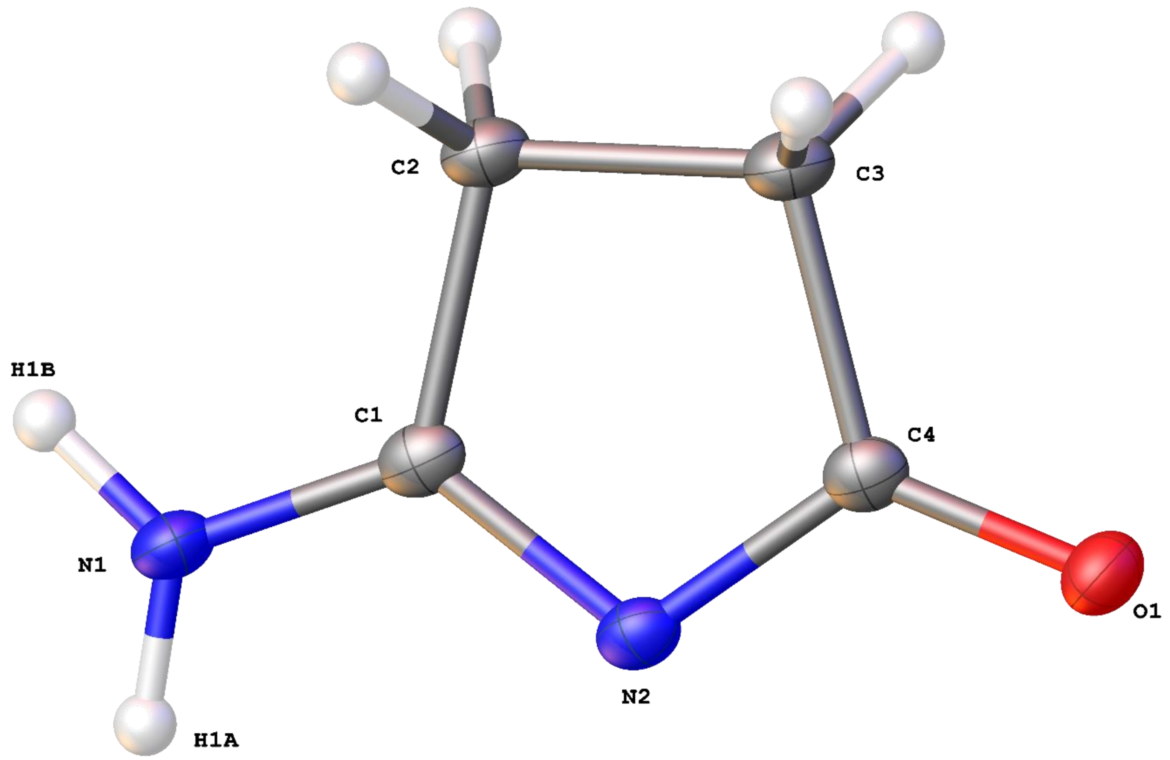

Figure S4. A molecular drawing of **3** shown with 50% probability ellipsoids.

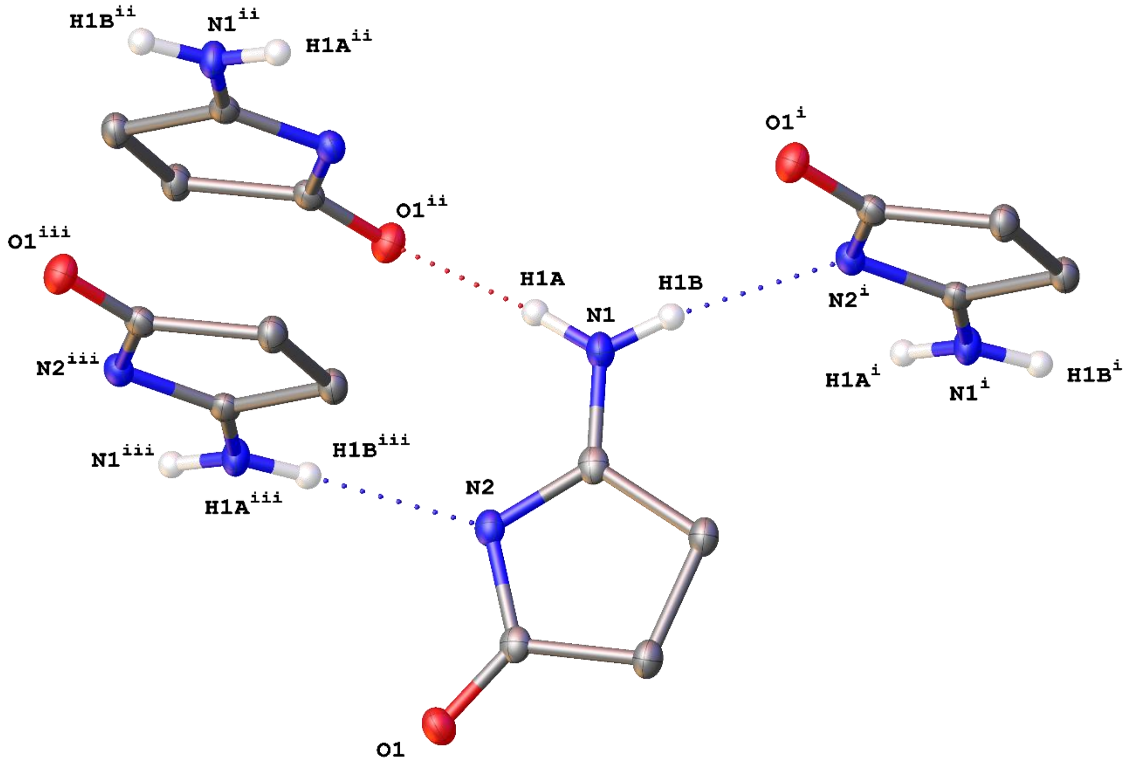

Figure S5. A molecular drawing of **3** shown with 50% probability ellipsoids with emphasis placed on the hydrogen bonding interactions. All H atoms not participating in hydrogen bonding interactions are omitted. [Symmetry code: i = -1/2+X,1/2-Y,-1/2+Z; ii = 3/2-X,-1/2+Y,3/2-Z; iii = 1/2+X,1/2-Y,1/2+Z.]

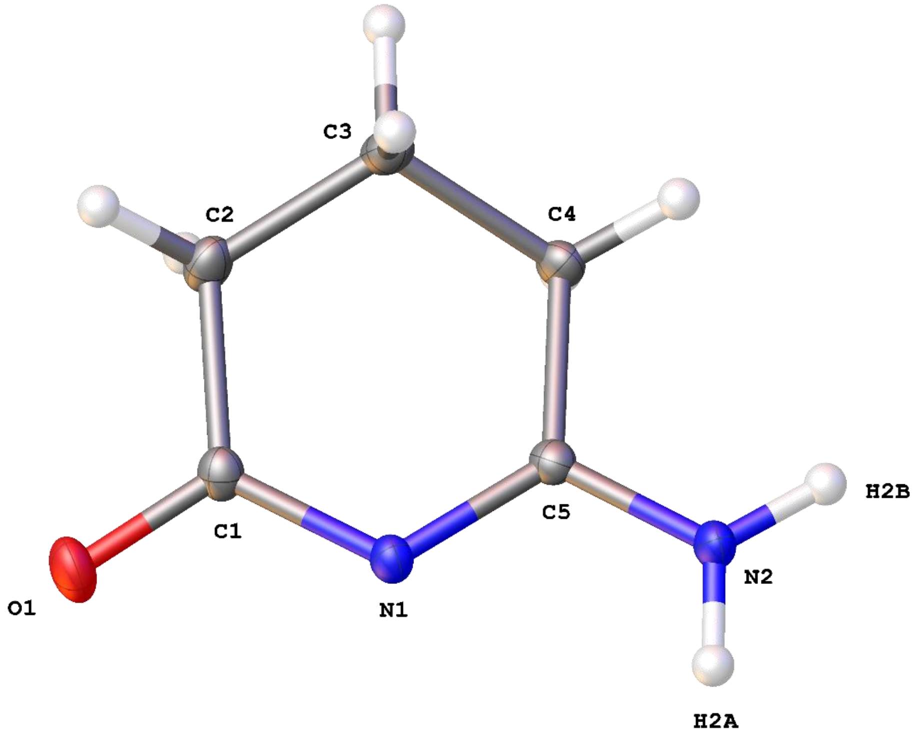

Figure S6. A molecular drawing of **4** shown with 50% probability ellipsoids.

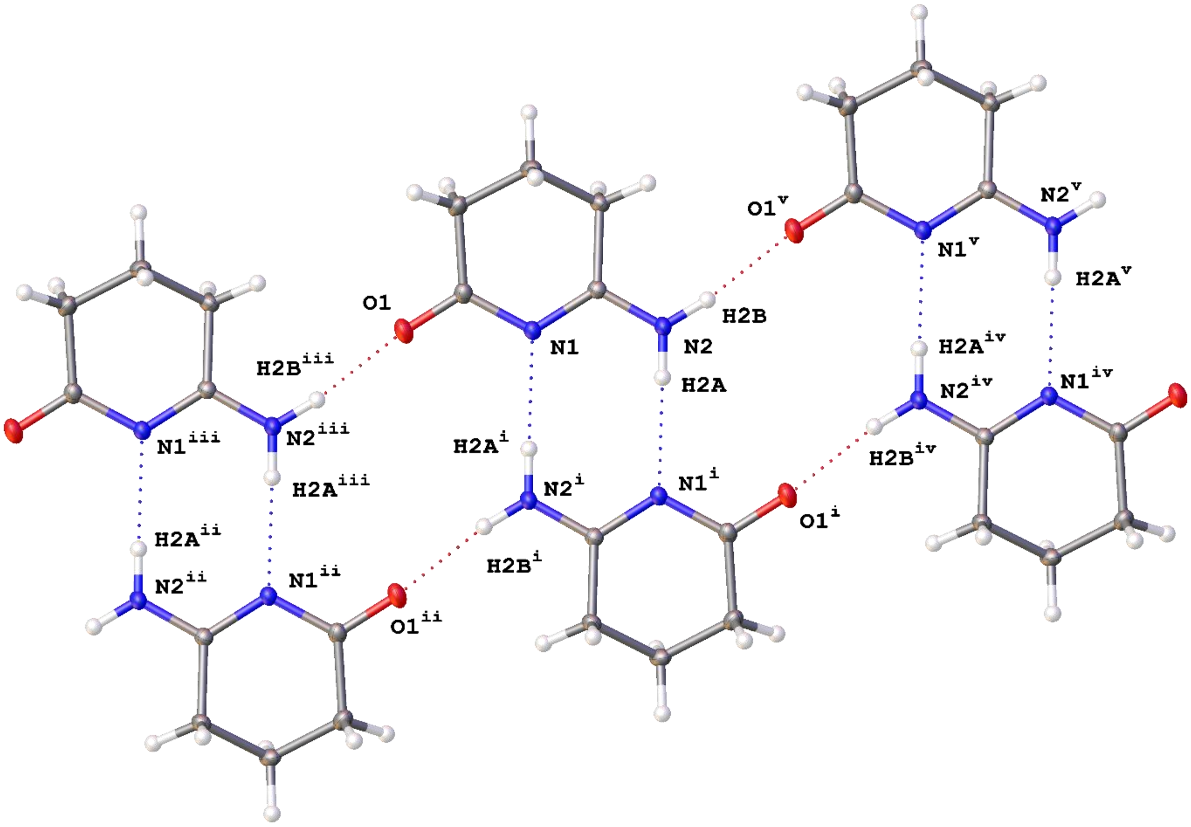

Figure S7. A molecular drawing of **4** shown with 50% probability ellipsoids. Emphasis is placed on the hydrogen bonding system. [Symmetry codes: i=-X,1-Y,1-Z; ii=-X,2-Y,1-Z; iii=+X,1+Y,+Z; iv=-X,-Y,1-Z; v=+X,-1+Y,+Z.]

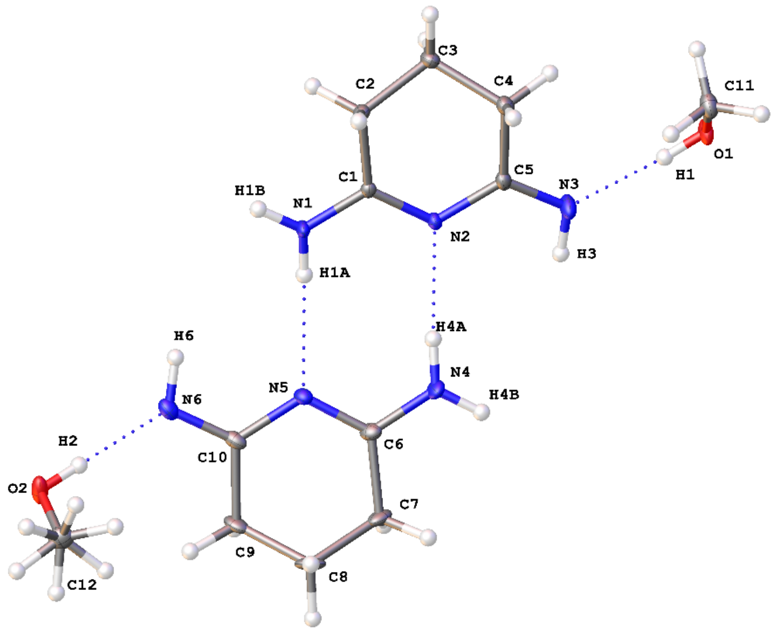

Figure S8. A molecular drawing of the major component of **2** shown with 50% probability ellipsoids.

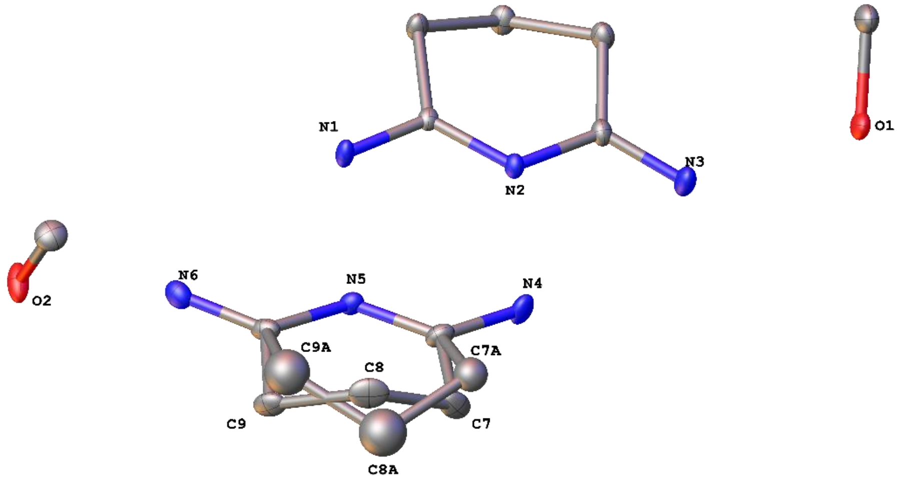

Figure S9. A molecular drawing of **2** shown with 50% probability ellipsoids. All Hydrogen atoms were omitted for clarity. Emphasis was placed on the disordered portion of the ring.
